# Human IgG4: a structural perspective

**DOI:** 10.1111/imr.12349

**Published:** 2015-10-26

**Authors:** Anna M Davies, Brian J Sutton

**Affiliations:** 1 Randall Division of Cell and Molecular Biophysics, King's College LondonLondon, UK; 2 Medical Research Council & Asthma UK Centre in Allergic Mechanisms of AsthmaLondon, UK

**Keywords:** immunoglobulin, antibody, IgG4, IgG1, Fc receptor

## Abstract

IgG4, the least represented human IgG subclass in serum, is an intriguing antibody with unique biological properties, such as the ability to undergo Fab-arm exchange and limit immune complex formation. The lack of effector functions, such as antibody-dependent cell-mediated cytotoxicity and complement-dependent cytotoxicity, is desirable for therapeutic purposes. IgG4 plays a protective role in allergy by acting as a blocking antibody, and inhibiting mast cell degranulation, but a deleterious role in malignant melanoma, by impeding IgG1-mediated anti-tumor immunity. These findings highlight the importance of understanding the interaction between IgG4 and Fcγ receptors. Despite a wealth of structural information for the IgG1 subclass, including complexes with Fcγ receptors, and structures for intact antibodies, high-resolution crystal structures were not reported for IgG4-Fc until recently. Here, we highlight some of the biological properties of human IgG4, and review the recent crystal structures of IgG4-Fc. We discuss the unexpected conformations adopted by functionally important Cγ2 domain loops, and speculate about potential implications for the interaction between IgG4 and FcγRs.

## Introduction

Of the four human IgG subclasses, IgG4 is the least abundant in serum at approximately 5% of total IgG (1). In contrast to IgG1, the structure and properties of which have been more extensively characterized, modified, and reviewed, the structure and biological functions of IgG4 are less well understood. Aalberse, Rispens and co-workers have described IgG4 as an ‘odd’ antibody due to its unique biological properties, and even considered this subclass to ‘break the rules’ in not conforming to conventional understanding of antibody structure (1,2).

Among human antibodies, IgG4 uniquely undergoes a process *in vivo*, termed Fab-arm exchange (FAE), in which bi-specific, functionally monovalent antibodies are created (2). This contributes to the anti-inflammatory properties of IgG4 and limits its ability to form immune complexes and activate complement (2–4). Furthermore, IgG4 is an attractive format for therapeutic monoclonal antibodies when effector function is undesired (5–7).

The involvement of IgG4 in disease is increasingly recognized. Elevated serum IgG4 levels, and IgG4 auto-antibodies directed against IgG (rheumatoid factors) and citrullinated proteins, are but some features of rheumatoid arthritis, an auto-immune, chronic inflammatory disease (8–11). Intriguingly, anti-IgG4 hinge antibodies, generated against cleaved antibody fragments under inflammatory conditions, such as those in rheumatoid arthritis, have been reported to form immune complexes and activate complement, which is suggested to ‘antagonize’ the anti-inflammatory properties of IgG4 (12). Furthermore, a spectrum of inflammatory diseases, affecting different organs, have now been classified under the term ‘IgG4-related disease’ in which IgG4 serum levels are often raised, and IgG4-positive plasma cell infiltrates are found. However, the contribution of IgG4 to disease pathogenesis is still not fully understood (13,14).

While recent evidence has also revealed a deleterious role for IgG4 in anti-tumor responses, through FcγRI ‘blockade’ (15), IgG4 plays a protective role in allergic disease by inhibiting mast cell degranulation (16), highlighting the importance of understanding its interaction with FcγRs.

Recently, high-resolution crystal structures for the IgG4 Cγ3 domain dimer and the IgG4-Fc region were reported (17–19), which not only provided insights into the phenomenon of FAE, but also revealed unexpected structural alterations in Cγ2 loop regions, with implications for C1q and FcγR binding.

In this review, we aim to bring together research from the fields of allergology, oncoimmunology, and structural biology, with IgG4 and its receptor interactions as the focus. We begin by providing an overview of IgG structure, and the range of proteins that engage the Fc region. We will then turn to some of the biological properties and functions of IgG4, before describing our recent X-ray crystallographic studies. In light of the unique structure of IgG4, we conclude with some speculative remarks about the molecular basis for the interaction between IgG4 and FcγRs.

## IgG antibody architecture

**Fig. 1:**
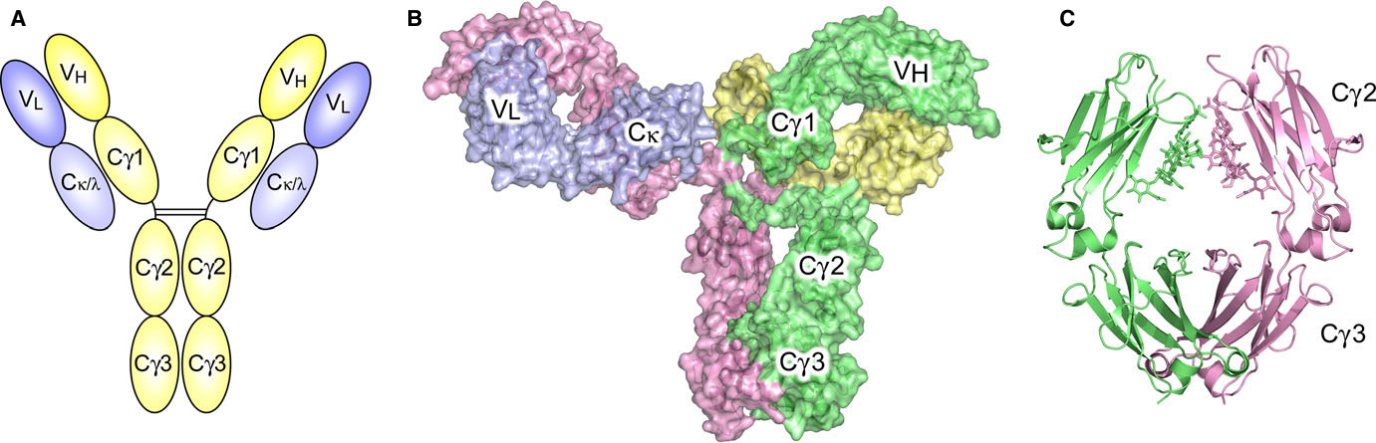
Overall IgG architecture. (A) Schematic of an IgG antibody. The Fc fragment, comprising Cγ2 and Cγ3 domains from the heavy chain, is connected to the Cγ1 domain of each Fab fragment through a hinge region. The sequence composition of the hinge, and number of inter-heavy chain disulfide bonds varies between the four human IgG subclasses. In this figure, two inter-heavy chain disulfide bonds are indicated for IgG1. The variable regions of the Fab fragment (V_H_ and V_L_) are responsible for antigen recognition. (B) Crystal structure of an intact human IgG1 antibody, solved at 2.7 Å resolution, reveals an asymmetric conformation (36). (C) Cartoon representation of the IgG-Fc fragment (50), showing the internal oligosaccharide moiety. The figure was prepared with PyMOL (166).

The four subclasses of human IgG antibodies (IgG1–4) are similar in their overall structure, in which the Fc region, comprising a pair of heavy-chain Cγ2 and Cγ3 constant domains, is connected to two Fabs, comprising V_H_ and Cγ1 (heavy chain) and V_L_ and Cκ/λ (light chain) domains, through a hinge (*Fig.* 1*A*). The Fc region is responsible for effector function, while the Fabs bind antigen through the variable domains. Although the constant regions display a high degree of sequence homology, variation in the length and sequence of the hinge region, and sequence variation in the Cγ2 and Cγ3 domains, further modulates the properties and effector functions of each subclass (20–25).

A biantennary oligosaccharide moiety, covalently attached to Asn297 in the Cγ2 domain, comprises two *N*-acetylglucosamine residues, and a branching mannose residue to which α(1–3) and α(1–6) ‘arms’ of mannose and *N*-acetylglucosamine residues are attached. The oligosaccharide moiety can additionally contain a fucose residue, attached to the first *N*-acetylglucosamine residue, and galactose and sialic acid residues attached to the α(1–3) and α(1–6) arms (26). The composition of the oligosaccharide moiety can modify the properties of IgG-Fc; for example, defucosylation increases the affinity for FcγRIIIa and enhances antibody-dependent cell-mediated cytotoxicity (ADCC) (27), sialylation reduces affinity for FcγRs and underlies the anti-inflammatory activity of IVIG therapy (28–32), while agalactosyl IgG is able to activate complement through the lectin pathway (33), and is correlated with disease activity in rheumatoid arthritis (34,35).

Due to their flexible nature, atomic resolution structural information for intact IgG antibodies is scarce. Crystal structures have been solved for only a few intact antibodies (36–39) and to date, a 2.7 Å resolution crystal structure of a human IgG1 antibody (*Fig.* 1*B*) is the highest resolution structure available (36). Instead, insights into the flexible nature of IgG antibodies, the disposition of the Fabs relative to the Fc region, and potential impact on effector function, have largely been provided by solution studies (40–48).

However, since the crystal structures reported by Deisenhofer in 1981 (49), over 60 structures have now been solved for human IgG-Fc (*Fig.* 1*C*), providing a wealth of information regarding the orientation of the Cγ2 domains, the structure of the internal oligosaccharide moiety, the structure of the lower hinge, and interactions with Fc receptors and other Fc-binding proteins. While the majority of these structures are for human IgG1-Fc (e.g. (50,49,51–71)), crystal structures have also been reported for human IgG2-Fc (72,73), human IgG4-Fc (18,19,74), as well as IgG-Fc from mouse (75–77), rat (78,79), and rabbit (80).

## IgG-Fc receptor-binding sites

IgG-Fc has a remarkable capability for engaging in different protein–protein interactions. IgG-Fc exploits two sites for receptor interactions, namely the lower hinge and hinge proximal Cγ2 domains to engage FcγRs, and the Cγ2–Cγ3 domain interface to engage FcRn and TRIM21.

Use of distinct sites for receptor engagement is also a key feature of the interaction between IgE and its two principal receptors, FcεRI and CD23, in which the Cε3 domains engage FcεRI, structurally homologous to the IgG Cγ2 domain and FcγRs, respectively, while the Cε3–Cε4 interface engages the C-type lectin receptor, CD23 (81–83). The interaction between IgE and its receptors is reviewed elsewhere in this volume (84).

Sialylation is suggested to facilitate binding of the C-type lectin, DC-SIGN (dendritic cell-specific intercellular adhesion molecule-3-grabbing non-integrin) to IgG-Fc, through adoption of a ‘closed’ conformation of the Cγ2 domains, in an analogous manner to the CD23 interaction at the IgE Cε3–Cε4 interface (85). Crystal structures for sialylated IgG1-Fc, in which the sialic acid residues are solvent exposed, differ in the position adopted by the Cγ2 domains relative to the Cγ3 domains (66,67), but as yet there is no crystal structure available for a DC-SIGN/IgG-Fc complex.

## IgG-Fc Cγ2–Cγ3 interface interacts with a diverse group of proteins

**Fig. 2:**
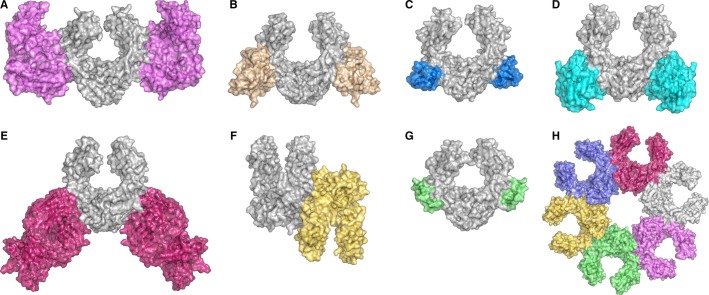
IgG utilizes the Cγ2–Cγ3 domain interface to interact with a variety of different proteins. (A) The neonatal receptor, FcRn (63). (B) HSV-1 (herpes simplex virus type I) gE-gI receptor (53). (C) Streptococcal protein G (52). (D) TRIM21 (tripartite motif-containing 21) (61). (E) Fab fragment from an IgM rheumatoid factor (74). (F) Fc–Fc interactions revealed by crystal packing (18). (G) Staphylococcal protein A (49). (H) Fc–Fc-mediated hexamer involved in complement activation (19,36,90). The figure was prepared with PyMOL (167).

The IgG-Fc Cγ2–Cγ3 interface is a promiscuous binding site, employing common ‘consensus’ residues to interact with a diverse range of proteins, and different structural motifs (62) (*Fig.* 2). The consensus residues, Met252, Ile253, Ser254, Asn434, His435, and Tyr436, are identical in IgG1, IgG2, and IgG4, but His435 and Tyr436 are substituted for Arg435 and Phe436 in IgG3. Two Fc receptors bind at the Cγ2–Cγ3 interface, namely FcRn and TRIM21. The pH-dependent interaction between IgG-Fc and the neonatal receptor, FcRn (*Fig.* 2*A*), is responsible for passive transfer of immunity from mother to fetus and control of serum half-life (63,78,79,86). On the other hand, the high-affinity, but pH independent, interaction with Tripartite motif-containing 21 (TRIM21) (*Fig.* 2*D*) provides a mechanism for intracellular antibody recognition in antiviral responses (61,77,87).

In addition to Fc receptors, the Cγ2–Cγ3 interface binds a variety of other proteins. In pathological conditions such as rheumatoid arthritis, the IgG-Fc fragment itself is the target of autoimmune antibodies (74). The consensus site at the Cγ2–Cγ3 interface (74) (*Fig.* 2*E*), and the Cγ3–Cγ3 interface (51), are both epitopes for IgM rheumatoid factors. Fc–Fc-mediated IgG interactions have also been documented in rheumatoid arthritis (88) and autoimmune pancreatitis (89), and crystal packing interactions in a number of IgG-Fc crystal structures (e.g. (18,50,68,69,71)) reveal contacts between residues from the consensus binding site (*Fig.* 2*F*). Furthermore, the IgG-Fc Cγ2–Cγ3 interface is also involved in forming Fc–Fc-mediated hexameric assemblies, associated with C1q binding (19,36,90) (*Fig.* 2*H*).

The IgG-Fc Cγ2–Cγ3 interface is also recognized by bacterial proteins such as staphylococcal protein A (49) (*Fig.* 2*G*) and streptococcal protein G (52) (*Fig.* 2*C*), which play a role in the host–microbe relationship (91), and is also exploited by the herpes simplex virus type 1 (HSV-1) gE-gI receptor, which mediates viral spread between cells (53) (*Fig.* 2*B*).

## FcγRs

**Fig. 3:**
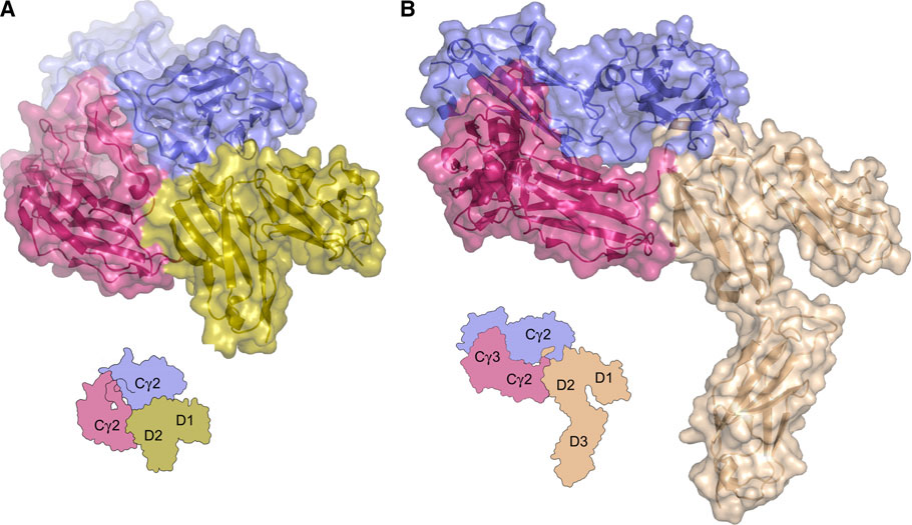
Overall structure of IgG1-Fc/FcγR complexes. (A) Crystal structure of the IgG1-Fc/FcγRIIIa complex (57). The domain arrangement is similar in the IgG1-Fc/FcγRIIa (60), FcγRIIb (65) and FcγRIIIb (56,59) complexes. (B) Crystal structure of the IgG1-Fc/FcγRI complex (55). In both panels, receptor domains are labeled D1-D3 and IgG-Fc domains Cγ2–Cγ3. The figure was prepared with PyMOL (167).

The three classes of human FcγRs and their sub-types, FcγRI (CD64), FcγRIIa/b/c (CD32a/b/c), and FcγRIIIa/b (CD16a/b), belong to the immunoglobulin superfamily, and are differentially expressed on leukocytes (92–97). The portion of the receptor which engages IgG-Fc is a transmembrane polypeptide chain with two extracellular domains (D1–D2) in FcγRII and FcγRIII, while FcγRI contains three extracellular domains (D1–D3) (*Fig.* 3). FcγRs display a range of affinities for the four IgG subclasses. In brief, FcγRI is a high-affinity receptor, able to bind monomeric IgG1, 3, and 4, whereas the low-affinity receptors, FcγRII and FcγRIII, engage surface-bound IgG, or IgG in the form of immune complexes, when the contribution from avidity effects can be realized. In contrast to IgG1, 3, and 4, IgG2 binds more weakly to FcγRs (98).

FcγR family members also differ in their mode of signal transduction: FcγRI and FcγRIIIa mediate signal transduction through association with the dimeric FcγR γ-chain, which contains an immunoreceptor tyrosine-based activation motif (ITAM), while FcγRIIa/c instead contain an ITAM, and FcγRIIb an immunoreceptor tyrosine-based inhibition motif, within the cytoplasmic portion of the IgG-binding polypeptide chain. In contrast, FcγRIIIb is attached to the membrane through a glycosyl-phosphatidylinositol anchor (92–97).

Activating receptors are responsible for effector functions such as antibody-dependent cellular phagocytosis (ADCP), ADCC, and the release of inflammatory mediators. Co-ligation of activating receptors with the inhibitory FcγRIIb receptor regulates these responses, while co-ligation of FcγRIIb and the B-cell receptor also downregulates B-cell activity (91,93,95–97,99,100). The interaction between IgG and FcγRs can play an important role in the mechanism of therapeutic monoclonal antibodies (101) and is thus a target for engineering. Properties such as enhanced, reduced, or selectively enhanced FcγR binding can be modified through engineering the IgG-Fc region, hinge, and oligosaccharide moiety (57,58,64,65,68,102,103).

A complex interplay of factors, including sequence differences in the IgG Cγ2 domain, length and sequence variation in the hinge, the disposition of the Fabs relative to the Fc region, glycosylation in both IgG and receptor, and sequence variation between receptors, all play a role in the interaction between IgG and FcγRs (21,32,44,103–106). Thus, for example, affinity constants (*K*_A_) for the interactions between IgG and the FcγRs range from undetectable levels for IgG4 and FcγRIIIb, to 6.5 × 10^7^ M^−1^ for IgG1 and FcγRI (97).

## IgG-Fc Cγ2 domain and lower hinge interact with Fcγ receptors

Crystal structures have now been solved for IgG1-Fc in complex with all three types of human FcγR (54–60,64,65). The interaction between IgG1-Fc and FcγRs has been reviewed elsewhere (e.g. (92,93,107–109)), but in light of two recent IgG1-Fc/FcγRI complex crystal structures (54,55), we will revisit this interaction once more. In this section, FcγRI, FcγRII, and FcγRIII residue numbers are according to Protein Data Bank entries 4X4M/FcγRI (54), 3RY6/FcγRIIa (60), and 1T89/FcγRIIIb (56), respectively, and will be used from hereon.

**Fig. 4:**
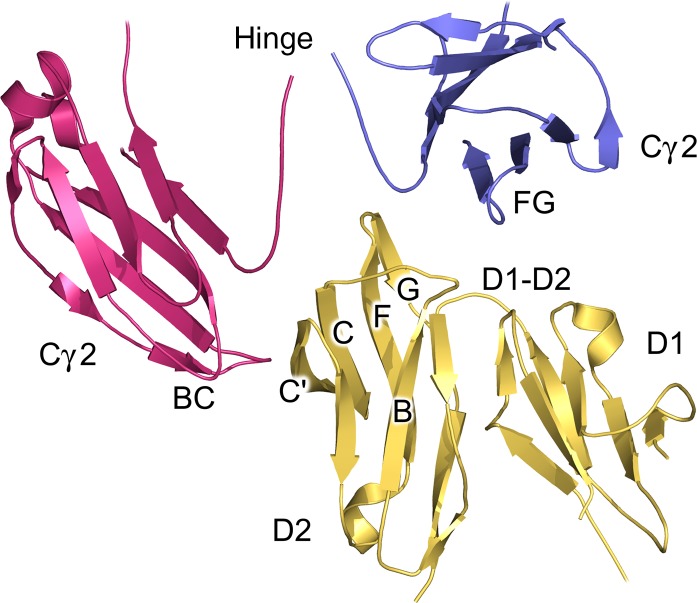
Sites of interaction in IgG1-Fc/FcγR complexes. A crystal structure for IgG1-Fc in complex with FcγRIIIa (58) is shown, although the interface is similar in all FcγRs. One IgG1-Fc Cγ2 domain (blue) interacts with the receptor (yellow) D1-D2 domain linker and D2 domain BC loop. The second IgG1-Fc Cγ2 domain (pink) interacts with the D2 domain C and C′ strands. The lower hinge contacts the D2 domain BC and FG loops. The figure was prepared with PyMOL (167).

In each, topologically similar FcγR complex, a single IgG1-Fc molecule engages one receptor, using the hinge proximal portion of each identical Cγ2 domain to create an asymmetric interaction at two distinct sites. One IgG1-Fc Cγ2 domain engages the FcγR D2 domain, the other Cγ2 domain interacts with the D1-D2 domain linker and D2 domain BC loop, while the lower hinge contacts the D2 domain C and C′ strands, and BC and FG loops (*Fig.* 4). There is no direct contact between IgG1-Fc and the FcγRI-III D1 domain, and the FcγRI D3 domain (*Figs* 3 *and* 4). The interaction between IgG1-Fc and FcγRI reveals the largest buried surface area between antibody and receptor, at over 2100 Å^2^ (54).

The interaction between IgG and FcγRs is structurally homologous to the interaction between the IgE-Fcε3-4 fragment and FcεRIα (81,83), but in IgE an extra Cε2 domain pair replaces the hinge found in IgG1. While the Cε2 domain pair does not directly contact FcεRIα, these domains not only stabilize the ‘molten globule-like’ Cε3 domains, reducing the entropic penalty upon FcεRIα binding, but promote closer contact between the Cε3 domains and receptor, burying a surface area of over 1800 Å^2^, to create a higher affinity interaction (*K*_A_ ∼ 10^10^ M^−1^) compared with IgG for FcγRs (81).

**Fig. 5:**
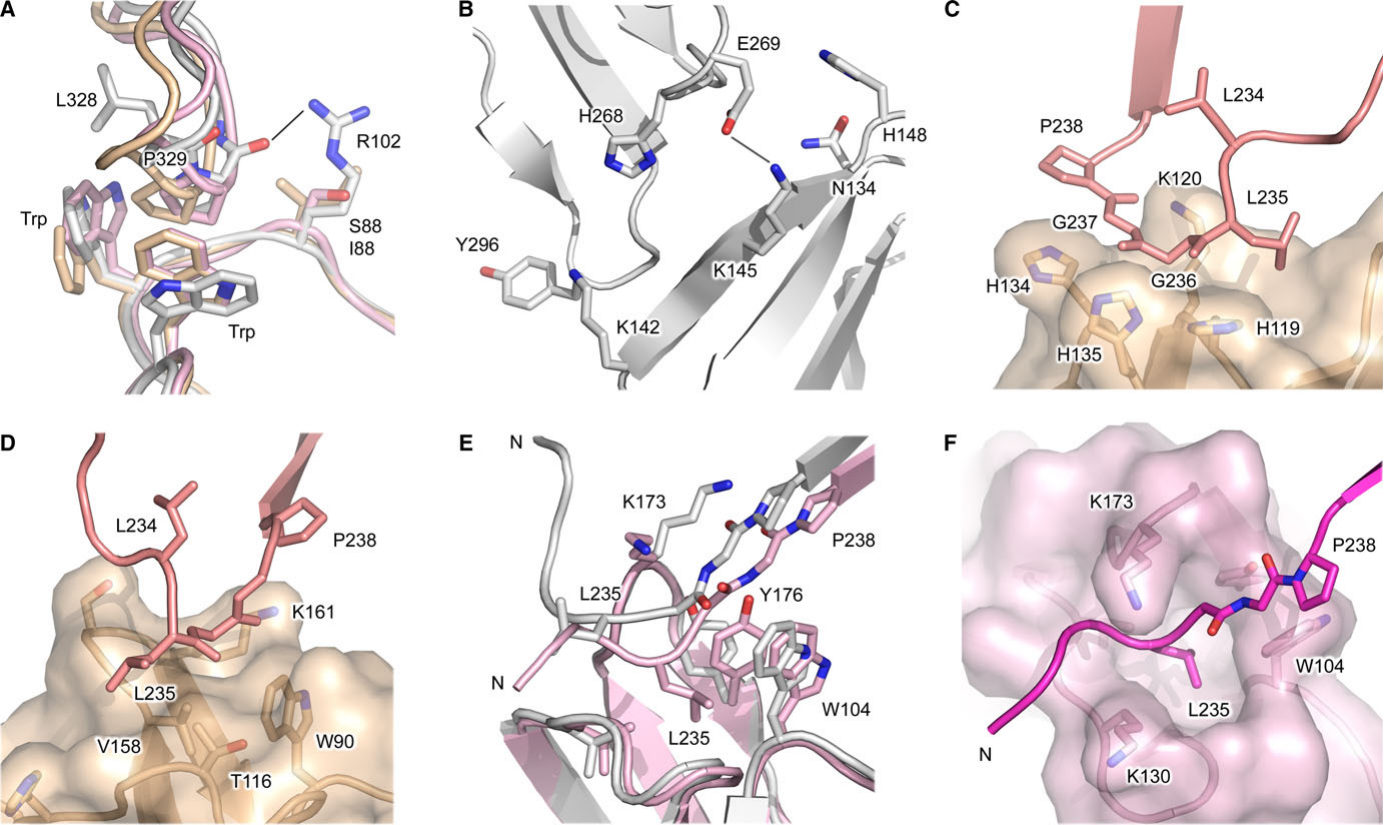
IgG1-Fc interactions with FcγRs. (A) The hydrophobic proline sandwich interaction, in which Pro329 from the Cγ2 FG loop interacts with two conserved tryptophan residues from the receptor. A position adjacent to the proline sandwich is a site of sequence variation, in which structurally equivalent residues are Arg102 in FcγRI (white) (54), Ser88 in FcγRII (pink) (65), and Ile88 in FcγRIII (beige) (58). In the FcγRI complex, Arg102 forms a hydrogen bond with the Pro329 backbone carbonyl group. (B) A second site of interaction involves the IgG1 Cγ2 domain BC and DE loops. In one IgG1-Fc/FcγRI complex (54), Lys142 from the receptor packs against Tyr296 (Cγ2 DE loop) while Lys145 from the receptor forms a hydrogen bond with Glu269 (Cγ2 BC loop). (C) In the IgG1-Fc/FcγRIIIa complex (58), the lower hinge from one IgG1 chain rests above a shallow groove created by His119, Lys120, His134, and His135 from the receptor. (D) In the IgG1-Fc/FcγRIIIa complex (58), the lower hinge from the second IgG1 chain is positioned above a depression created by Thr116, Ala117, Val158, and Lys161 from the receptor. (E) The position of the lower hinge differs in the two IgG1-Fc/FcγRI complexes. In one structure (white) (54), the lower hinge adopts a conformation akin to that in FcγRII and FcγRIII complexes, while in another structure (pink) (55), the hinge points away from the Fc region. (F) In one IgG1-Fc/FcγRI complex (55), Leu235 from the lower hinge occupies a hydrophobic pocket on the receptor. The figure was prepared with PyMOL (167).

In every crystal structure solved for IgG1-Fc in complex with an FcγR, the IgG1-Fc FG loop from one of the two identical Cγ2 domains contacts the receptor D1-D2 domain linker and D2 BC loop through a hydrophobic ‘proline sandwich’ interaction, in which Pro329 from IgG is positioned between two tryptophan residues from the receptor (54–60,64,65) (*Fig.* 5*A*). This structurally conserved mode of interaction is also found between the IgE Cε3 domain and FcεRI receptor (81,83). While the two tryptophan residues are invariant among the FcγRs, sequence variation is found at a structurally equivalent residue adjacent to the proline sandwich, and is arginine in FcγRI (Arg102), serine in FcγRII (Ser88), and isoleucine in FcγRIII (Ile88). In the FcγRI structures, Arg102 forms a hydrogen bond with the backbone carbonyl atom of Pro329 from the IgG1-Fc Cγ2 FG loop (54,55) (*Fig.* 5*A*).

On the other hand, the second site of interaction, between the other IgG1-Fc Cγ2 domain and receptor D2 domain, is characterized by hydrogen bonds and salt bridges, in addition to van der Waals interactions. In all structures, residues from the receptor D2 C and C′ strands interact with the IgG1-Fc Cγ2 BC and DE loops. Unlike the conserved tryptophan residues which create the proline sandwich interaction, the receptor D2 C and C′ strands display greater sequence variation between residues that contact IgG1-Fc. For example, a structurally equivalent residue from the C strand is Asn134 in FcγRI and Lys120 in FcγRII and FcγRIII. Structurally equivalent C′ strand residues which differ include Ala143 in FcγRI / Ser129 in FcγRII / Gly129 in FcγRIIIa / Asp129 in FcγRIIIb, positioned close to Asn297, the site of oligosaccharide attachment in IgG, and His148/134 in FcγRI and FcγRIIIa/b, respectively, and Arg134 in FcγRIIb, which is also the site of a Arg/His polymorphism in FcγRIIa [also referred to in the literature as the Arg131His polymorphism (93,98)]. On the other hand, C′ strand residues Lys142 (FcγRI)/128 (FcγRII and FcγRIII), positioned close to Tyr296 from the IgG1 Cγ2 DE loop, and Lys145 (FcγRI)/131 (FcγRII and FcγRIII), which can form a hydrogen bond with Glu269 from the IgG1 Cγ2 BC loop, are invariant among human FcγRs. (*Fig.* 5*B*). We note there is a difference between the FcγRI complex structures (54,55), involving a *cis*/*trans* proline isomerization in the IgG1-Fc Cγ2 BC loop that engages the FcγRI D2 domain, which will be discussed in further detail later.

The IgG1-Fc lower hinge is typically better ordered in FcγR complex structures, compared with those for the Fc fragment alone. The overall position of the hinge varies, but the lower hinge is generally similar in conformation (*Fig.* 4). The receptor contacts the lower hinge at two sites. At the first site, the receptor D2 domain C and C′ strands interact with the lower hinge from one IgG1-Fc chain (*Fig.* 4). In the FcγRIII complex, Gly236 and 237 from the lower hinge rest in a shallow groove created by His119, Lys120, His134, and His135 (*Fig.* 5*C*). The hinge position is comparable in the FcγRI structure, in which Gly236 and Gly237 rest above a groove created by Tyr133, Asn134, His148, and Trp149. The FcγRIIb complexes contain IgG1-Fc fragments with mutations in their hinge region, thus the interaction between wildtype IgG1-Fc and this receptor is unknown (65). However, in the FcγRIIa structure, His119, 134, and 135 are replaced by Val119, Arg134, and Leu135, respectively, and the lower hinge conformation differs, positioned instead above Val119.

At the second site of the lower hinge/receptor interaction, the receptor D2 BC and FG loops interact with the other IgG-Fc chain (*Fig.* 4). While the D2 FG loop sequence is not conserved among the three different FcγR classes, the overall loop structure is similar in FcγRII and FcγRIII complexes. In the FcγRIII complex, Leu235 and Gly236 from the lower hinge are positioned above a depression created by Thr116, Ala117 (D2 BC loop), Val158 and Lys161 (D2 FG loop) (*Fig.* 5*D*). The lower hinge adopts a similar conformation in the FcγRIIb structure, in which Lys116, Pro117, and Ile158 create a depression. In both FcγRII and FcγRIII complex structures, a tryptophan residue from the proline sandwich interface borders the interface with the lower hinge. Position 158 from the D2 domain FG loop (FcγRII/FcγRIII numbering) is not only a site of sequence variation between different types of FcγR (93,98) but also the site of a Val/Phe polymorphism in FcγRIIIa.

The two recent FcγRI complex structures each paint a different picture of the interaction between IgG1-Fc, and the receptor D2 FG loop, which is one residue shorter than its counterparts in FcγRII and FcγRIII. In the IgG1-Fc/FcγRI complex solved by Lu *et al*. (54), the hinge adopts a position more akin to those found in FcγRII and FcγRIII complex structures, facing away from the D2 domain, toward the Fc region. The hinge is more disordered in the FcγRI complex solved by Kiyoshi *et al*. (55), but points away from the Fc region. The orientation of the N-terminal hinge residues implies that the Fab fragments could adopt significantly different positions in these complexes (*Fig.* 5*E*).

The hinge positions in the FcγRI structures can be attributed to different interactions between the lower hinge, particularly Leu235, and the receptor D2 FG loop, which in FcγRI adopts a different conformation compared with the FcγRII and FcγRIII structures. The overall conformation of the FcγRI D2 FG loop is similar in both complexes, as well as the unliganded receptor (32). However, in the structure solved by Kiyoshi *et al*. (55), Leu235 from the lower hinge occupies a hydrophobic pocket created predominantly by Trp104 (D1-D2 linker), Lys130 (BC loop), Val132 (BC loop), Lys173 (FG loop), and Tyr176 (FG loop) from the receptor (*Fig.* 5*F*). On the other hand, in the structure solved by Lu *et al*. (54), Lys173 forms a salt bridge with Asp265 from the IgG1 Cγ2 domain, while Leu235 is solvent exposed (*Fig.* 5*E*).

## Affinity of IgG4 for FcγRs

Numerous studies have clearly established that the four IgG subclasses display a range of binding affinities for FcγRs (e.g. (21,32,55,106,110–112)). A comprehensive investigation was conducted by Bruhns *et al*. (97) in 2009. IgG1 displayed a range of binding affinities for FcγRs: the affinity constant (*K*_A_) for FcγRI was the highest at 6.5 × 10^7^ M^−1^, those for FcγRIIa^Arg/His134^ and FcγRIIIa^Phe/Val158^ were lower at 1.2–5.2 × 10^6^ M^−1^, while values for FcγRIIb and FcγRIIIb were the lowest, at 1.2–2.2 × 10^5^ M^−1^ (98). By contrast, IgG4 lacked the range of binding affinities observed for IgG1 and FcγRs. IgG4 bound FcγRI with a *K*_A_ of 3.4 × 10^7^ M^−1^, the same order of magnitude as that for IgG1, but those for FcγRIIa/b and FcγRIIIa were only the same order of magnitude as the lowest IgG1 values (1.7–2.5 × 10^5^ M^−1^), while binding to FcγRIIIb was not detected (98).

## Fab-arm exchange – an intriguing property

**Fig. 6:**
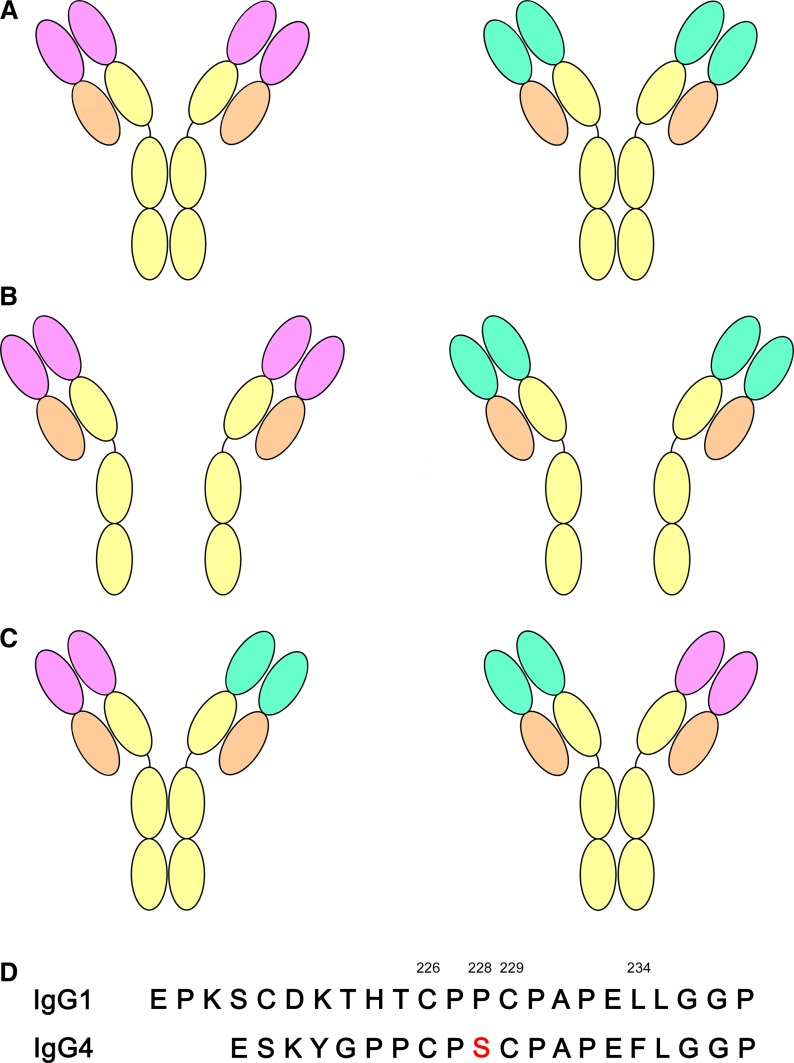
Fab-arm exchange. (A) Two intact IgG4 antibodies with different specificities are indicated by different colors for the variable domains. (B) Antibodies separate into ‘half-molecules’, each comprising one heavy and one light chain. (C) Half-molecules recombine to form bi-specific antibodies. (D) Amino acid sequence of the IgG1 and IgG4 hinges (168). In IgG4, position 228 is serine, compared with proline in IgG1. Inter-chain disulfide bonds form between Cys226 and Cys229 in IgG1, while intra-chain disulfide bonds can form in IgG4.

Of the human IgG subclasses, IgG4 has the unique ability to undergo FAE (*Fig.* 6). The process involves separation of the two IgG4 heavy chains to form ‘half-molecules’ comprising just one heavy and light chain (*Fig.* 6*A, B*). Half-molecules of any specificity can recombine to create bi-specific antibodies (2) (*Fig.* 6*C*). Two determinants enable IgG4 to undergo FAE, namely the core hinge and the Cγ3–Cγ3 domain interface (2,22,23), affecting both covalent and non-covalent interactions between the two heavy chains. In IgG1, which does not undergo FAE, the core hinge forms two inter-heavy chain disulfide bonds, formed by Cys226 and Cys229. While the IgG4 core hinge contains equivalent cysteine residues, it also contains a Pro228Ser substitution (*Fig.* 6*D*), which is suggested to promote a more flexible hinge region, leading to the formation of intra- rather than inter-heavy chain disulfide bonds (2,112). While the Fabs stabilize IgG4 antibodies with inter-heavy chain disulfide bonds (114), up to 73% of IgG4 molecules have been reported to lack this covalent interaction (115). In a recent study using human serum, it was found that up to 33% of IgG4 molecules were κ/λ light-chain hybrids, which was attributed to FAE, and the authors acknowledged that additional FAE could also have occurred between antibodies containing the same light-chain type (116).

The Cγ3–Cγ3 domain interface contains a critical residue for FAE at position 409. In IgG1, a lysine is found at this position, but IgG4 contains a Lys409Arg substitution, which weakens the Cγ3–Cγ3 domain interaction (23,117). While the structural consequences of the Lys409Arg substitution have been elucidated (17), the structure of the IgG4 hinge, and the mechanism by which the Fabs stabilize an intact IgG4 hinge, is unknown.

The ability of IgG4 to undergo FAE *in vivo*, creating bi-specific, functionally monovalent antibodies is suggested to contribute to its anti-inflammatory properties by limiting immune complex formation (2,22).

## IgG4 as a therapeutic antibody

IgG4 is considered to be an attractive therapeutic monoclonal antibody format when effector function is undesired (5–7). For example, pembrolizumab and nivolumab, anti-PD-1 (programmed death-1) IgG4 cancer therapeutics, both approved in the USA in 2014, inhibit the interaction between the immunoinhibitory T-cell PD-1 receptor and its ligands, but do not elicit ADCC or complement-dependent cytotoxicity (118–120).

Stabilization of the core hinge to prevent FAE is a design consideration for therapeutic IgG4 antibodies (121), as those without the core hinge Ser228Pro mutation, which abrogates FAE (122), have been demonstrated to undergo FAE with endogenous IgG4 (121). Both pembrolizumab and nivolumab contain the Ser228Pro mutation in their hinge. On the other hand, natalizumab, an anti-α4-intergrin IgG4 therapeutic used in the treatment of multiple sclerosis and Crohn's disease, contains a wildtype hinge sequence and undergoes FAE *in vivo* (121,123), but the authors of one study noted that there were no data to indicate that FAE had any consequences for the clinical effects of this therapeutic antibody (123).

While the therapeutic monoclonal antibody market is dominated by the IgG1 subclass, a number of IgG4 antibodies, with wildtype or stabilized hinges, are currently in clinical trials, including anti-IL-5 reslizumab for the treatment of asthma (124), anti-IL-17 ixekizumab for the treatment of psoriasis (125), anti-IL-13 tralokinumab for the treatment of asthma (126), and anti-CD22 inotuzumab ozogamicin, an antibody-drug conjugate (127) for the treatment of acute lymphoblastic leukemia, which highlights the suitability of IgG4 for therapeutic purposes.

## IgG4 – a protective role in allergy

IgE plays a central role in the allergic cascade, in which crosslinking by allergen of FcεRI-bound IgE on the mast cell and basophil cell surface triggers degranulation (125). The T_H_2 response, which controls B-cell class switching to both IgG4 and IgE, requires IL-4 or IL-13 cytokines. However, in a ‘modified T_H_2 response’, IL-10 production in the presence of IL-4 drives class switching to IgG4, without IgE production (1,129–131).

In addition to FcεRI, mast cells and basophils express the FcγRII receptor (93,95,132). While co-aggregation of FcγRIIa can induce mast cell degranulation (94,133,134), co-aggregation of FcεRI and FcγRIIb by IgE and IgG immune complexes can negatively regulate mast cell activation (94,135–137). Another protective mechanism which could inhibit mast cell degranulation is competition with IgE for allergen by a ‘blocking antibody’ (136,138,139).

While IgG4 is the least represented IgG subclass in serum, at less than 5% of total IgG, IgG4 levels can reach 75% of total IgG after chronic exposure to antigen (1,140). Elevated serum antigen-specific IgG4 levels are also associated with successful allergen-specific immunotherapy in the treatment of allergic disease (138).

A grass pollen-specific IgG4 antibody isolated from a patient who had received immunotherapy blocked the interaction between allergen and IgE and inhibited basophil activation (138). Furthermore, IgE-facilitated antigen presentation by B cells, which promotes allergic inflammation, and first requires engagement of membrane CD23 by IgE-allergen complexes, was also inhibited. FAE, and the limited potential for IgG4 to form immune complexes, could contribute to this ‘blocking’ ability (141). In a recent study of peanut allergy, serum from patients who were sensitized, but peanut-tolerant, or who had received oral immunotherapy, contained peanut-specific IgG4 antibodies which inhibited mast cell and basophil activation by peanut-specific IgE, although the mechanism by which IgG4 exerted its protective effects (as a blocking antibody or through co-aggregation of FcεRI and FcγRIIb) was not established (16).

The mechanism by which IgG4 exerts a protective role in allergic disease clearly merits further investigation, and it is important to note that of all IgG subclasses, IgG4 has the highest affinity for the inhibitory receptor FcγRIIb (98), which could have implications for the inhibition of mast cell/basophil activation.

## IgG4 – a deleterious role in cancer

The role of B-cell responses in cancer is not fully understood. However, infiltration of tumors by B cells, organized into tumor-associated lymphoid structures, is associated with a positive prognosis. Within these lymphoid structures, B cells are able to undergo class-switch recombination and somatic hypermutation, and mount anti-tumor-specific antibody responses (142–144).

IgG4-positive plasma B-cell infiltrates have been reported in cancers such as extrahepatic cholangiocarcinoma (145), pancreatic cancer (146), and malignant melanoma (15). The role of IgG4 in cancer is poorly understood; however, a recent study has provided significant new insights. In their study of malignant melanoma, Karagiannis *et al*. (15) revealed that tumor-specific IgG4 was produced locally in the tumor microenvironment, and that IL-4 and IL-10 expression was enhanced. The authors also discovered that while tumor-specific IgG1 antibodies, directed against the chondroitin sulfate proteoglycan 4 (CSPG4) tumor antigen (147), were able to facilitate monocyte-mediated ADCC and ADCP of tumor cells, tumor-specific IgG4 was unable to elicit the same response. Furthermore, when translated to an *in vivo* model, tumor-specific IgG1 was able to restrict tumor growth, while IgG4 could not. IgG4 was discovered to impair IgG1-mediated cytotoxicity, and activation of downstream signaling cascades, through competition for FcγRI binding, in effect ‘blockading’ the receptor (15).

It is intriguing that despite affinities for FcγRI which are of the same order of magnitude (98), IgG1 and IgG4 antibodies produce different anti-tumor responses in malignant melanoma. The molecular mechanism for FcγRI blockade by IgG4 is currently unknown, and whether receptor blockade is a common feature in cancers characterized by IgG4-positive tumor infiltrates remains to be determined.

## Recent insights into the structure of human IgG4-Fc

In 1997, the first crystal structure for human IgG4-Fc was reported by Sutton and co-workers, in a complex with the Fab fragment from an IgM rheumatoid factor (RF-AN), revealing an overall conformation similar to that previously reported for IgG1-Fc (75) (*Fig.* 2*E*). Four years earlier, it had been suggested that certain sequence differences between IgG1 and IgG4, such as Pro331Ser, might cause structural changes to the IgG4 Cγ2 domain loop structure (148). However, the low (3.15 Å) resolution of the IgG4-Fc/rheumatoid factor complex, and the disordered nature of a significant portion of the Cγ2 domain, precluded any investigation into subtle structural differences between the two subclasses. While over 60 structures have been reported for human IgG1-Fc, until recently, the IgG4-Fc/RF-AN complex was the sole crystal structure available for IgG4-Fc.

### Cγ3 domain dimer

The ability of IgG4 to undergo FAE (2,22,121) re-kindled our interest in further understanding this subclass, by determining whether high-resolution crystal structures could provide insights into the phenomenon. Together with our collaborators, we first turned our attention to the Cγ3 domain, and residue 409 at the Cγ3–Cγ3 interface, the identity of which is critical for FAE. In IgG1, which does not undergo FAE, residue 409 is lysine, whereas in IgG4, the equivalent residue is arginine (23).

**Fig. 7:**
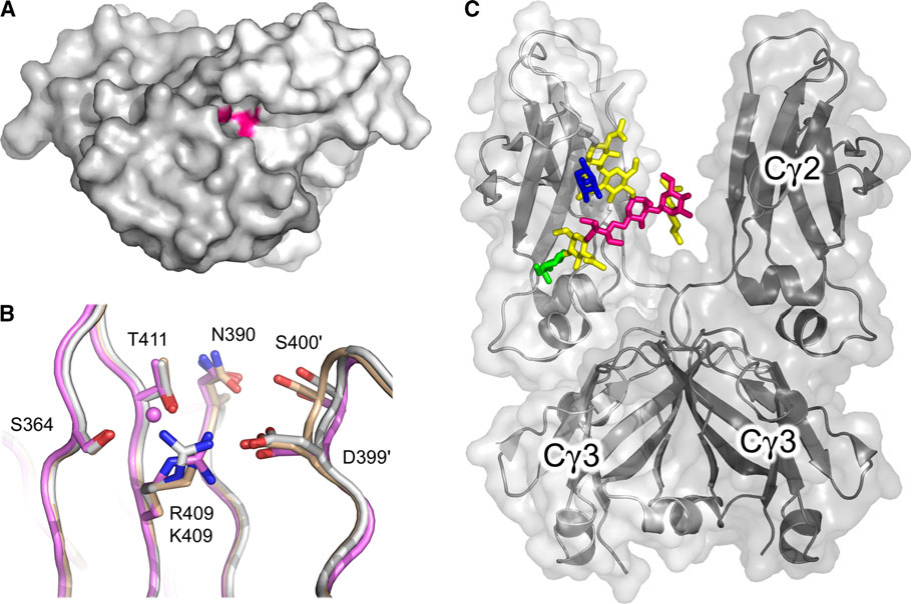
IgG4-Fc structure. (A) Crystal structure of the IgG4 Cγ3 domain dimer (17). The two Cγ3 domains are colored in light and dark gray, and the position of Arg409 at the Cγ3–Cγ3 interface is colored pink. (B) Arg409 adopts two conformations at the Cγ3–Cγ3 interface. One conformation (pink) is compatible with a conserved network of four water molecules, of which one is shown. The second conformation (white) disrupts the conserved network. IgG1-Fc, in which residue 409 is lysine, is colored beige. Residues from the second Cγ3 domain are indicated by a prime symbol. (C) Overall structure of IgG4-Fc (18). The two chains are colored in light and dark gray. The oligosaccharide moiety from one chain is colored as follows: *N*-acetylglucosamine, yellow; mannose, pink; fucose, blue; galactose, green. The figure was prepared with PyMOL (167).

We solved the crystal structure of the IgG4 Cγ3 domain dimer to 1.8 Å resolution (17) (*Fig.* 7*A*). While comprising just a portion of the Fc region, this crystal structure provided the first high-resolution structural information for IgG4, and the first structural insights into FAE. In IgG1, the conformation of Lys409 is generally conserved, and a network of water molecules mediates inter-domain hydrogen bonds. Substitution of lysine for arginine in IgG4 disrupts the conserved water molecule network, and reduces the contact area between the two Cγ3 domains at the edge of the interface, as a result of an altered Cγ3 DE loop position (*Fig.* 7*B*). In IgG1, Ser400 from the DE loop is able to form a hydrogen bond with Asn390 from the other Cγ3 domain, effectively ‘closing’ a groove at the interface edge, but in IgG4 this interaction is prevented by the bulkier arginine residue. The weakening effect of Arg409 on the Cγ3–Cγ3 interface is consistent with the requirement of the Cγ3 domain pair to dissociate in FAE (17,23,114).

We wondered whether Arg409 was conformationally flexible, and if so, what would be the effects on the Cγ3–Cγ3 interface? The conformation of Arg409 in the Cγ3 domain dimer structure differed from that modeled in the earlier IgG4-Fc structure. Furthermore, the low-resolution data had also precluded inclusion of water molecules. A high-resolution crystal structure for IgG4-Fc was thus warranted.

### IgG4-Fc

Seventeen years after the first, and only, IgG4-Fc structure was solved, we reported the first high-resolution crystal structures for both recombinant (1.9 Å) and serum-derived (2.35 Å) human IgG4-Fc, providing a level of detail not available in the earlier, low-resolution structure (18).

The overall IgG4-Fc topology resembled that of other IgG-Fc structures (*Fig.* 7*C*). In contrast to the low-resolution IgG4-Fc structure, a substantial portion of the oligosaccharide moiety was modeled in both high-resolution structures. In addition to a complex biantennary core, with an inter-chain hydrogen bond between α(1-3) branch mannose residues, a fucose residue attached to the first *N*-acetylglucosamine residue and a galactose residue on the α(1-6) branch adopted similar positions to those in human IgG1-Fc structures. (50,61) (*Fig.* 7*C*).

The two new IgG4-Fc structures added a further piece to the FAE puzzle, as we discovered that Arg409 was indeed able to adopt two different conformations at the Cγ3–Cγ3 interface. In addition to the interface-weakening conformation found in the Cγ3 domain dimer structure, a second conformation was observed which did not disrupt the conserved water molecule network (*Fig.* 7*B*), and was more akin to the IgG1 Cγ3–Cγ3 interface. Together, these structures provided evidence for a dynamic Cγ3–Cγ3 interface in IgG4.

### Cγ2 domain loops are conformationally altered in IgG4-Fc

**Fig. 8:**
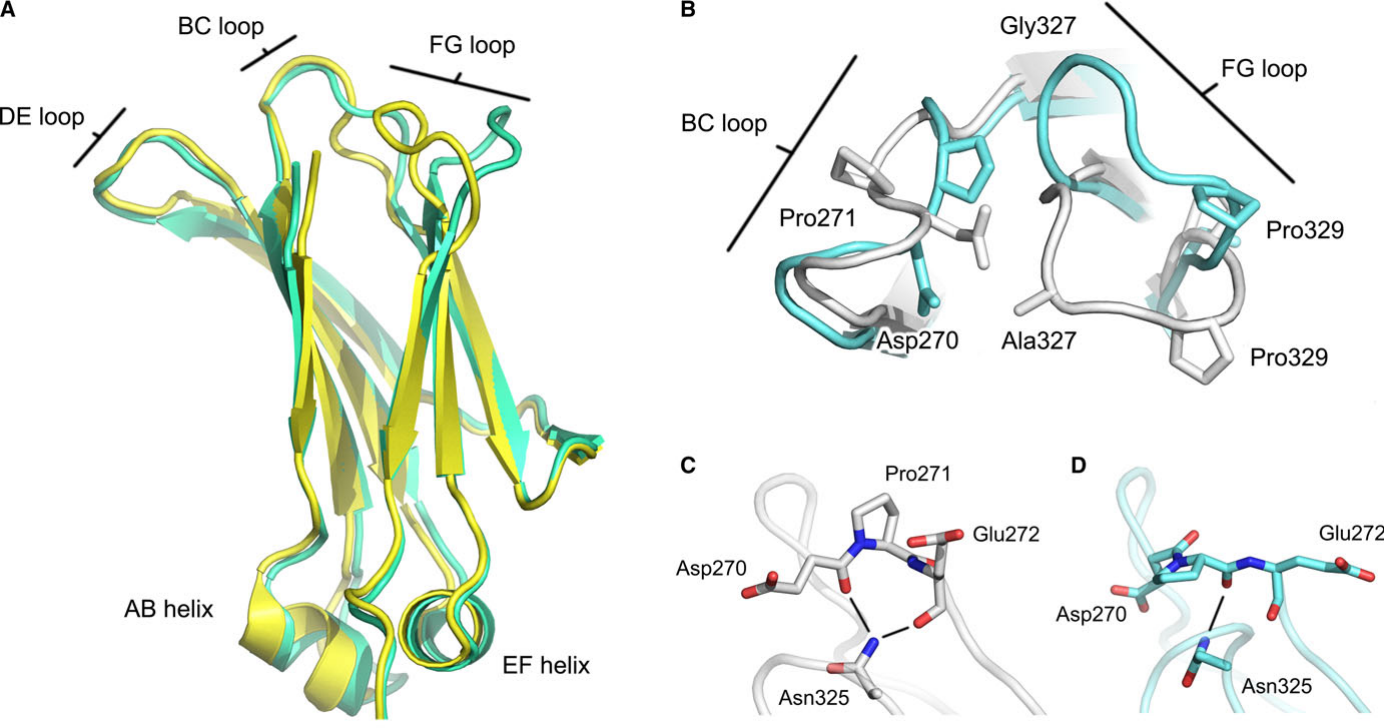
Conformational differences between IgG1 and IgG4 Cγ2 domain loops. (A) Overall structure of the IgG1 (yellow) (58) and IgG4 (green) (18) Cγ2 domain. While the overall domain structure is conserved, the conformation of BC and FG loops is different, and in IgG4, the FG loop folds away from the Cγ2 domain. (B) In IgG4 (blue) (18), Cα atoms for residues 327 (Gly in IgG4, Ala in IgG1) and Pro329 from the FG loop differ from their positions in IgG1 (white) (55) by approximately 6.7 and 9.9 Å, respectively. The positions of Asp270 and Pro271 from the BC loop are also significantly altered. (C) In IgG1 (55), the Asn325 side chain is able to form hydrogen bonds, indicated by black lines, with carbonyl oxygen atoms of Asp270 and Glu272 from the BC loop. (D) In IgG4 (18), Asn325 could instead form a hydrogen with the carbonyl oxygen atom of Pro271. The figure was prepared with PyMOL (167).

**Fig. 9:**
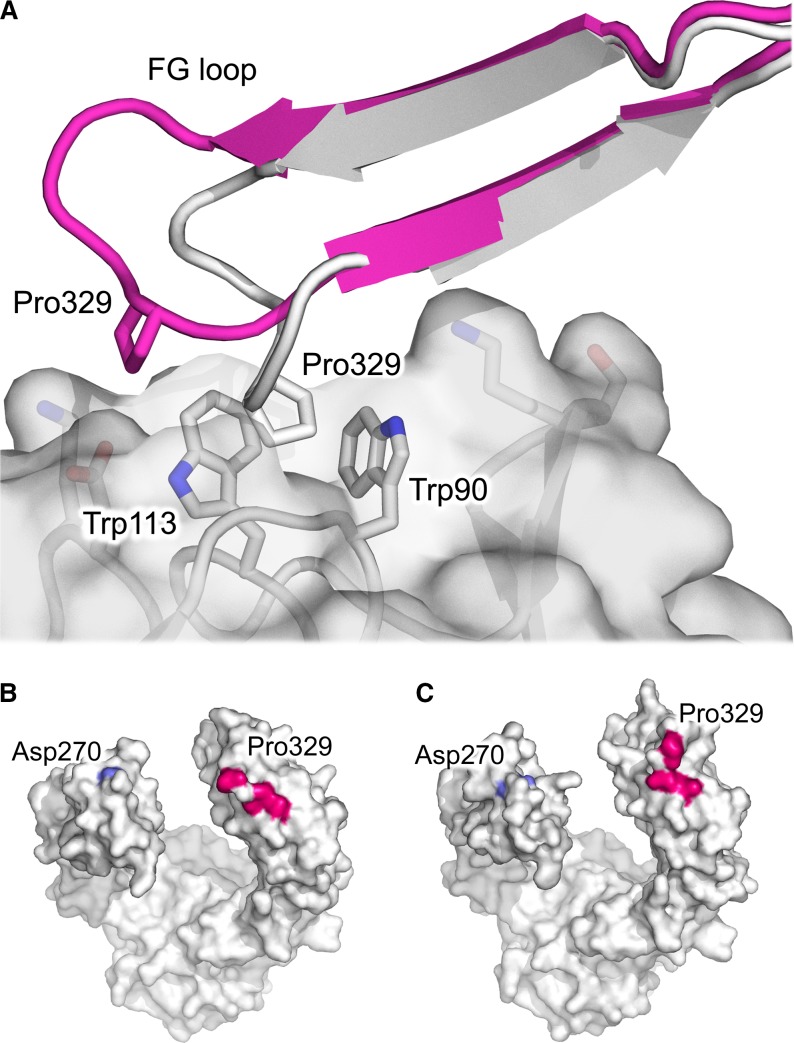
Disrupted FcγR and C1q binding sites in IgG4. (A) In all IgG1-Fc/FcγR complex structures, a hydrophobic ‘proline sandwich’ interaction forms between Pro329 from the IgG Cγ2 domain FG loop and two tryptophan residues from the receptor. The interaction between IgG1-Fc and FcγRIIIa is shown in gray (58). In IgG4-Fc (pink), the unique Cγ2 FG loop conformation would disrupt this conserved interaction (18). (B) Residues from IgG1-Fc (55) which are important for C1q binding are colored according to a model for the interaction between IgG1-Fc and C1q (164). The positions of Asp270 and Pro329 are indicated. (C) In IgG4-Fc (18), the positions of C1q binding residues are altered. The figure was prepared with PyMOL (167).

Crucially, the Cγ2 domains were ordered in both high-resolution IgG4-Fc structures. Although some structural alteration to the IgG4 Cγ2 domain loops had been envisaged two decades previously (148), the extent of the conformational differences between IgG4-Fc and IgG1-Fc structures was completely unexpected (*Fig.* 8*A*). In both IgG4-Fc structures, the Cγ2 FG loop (residues 325–330) adopted a different structure, and the positions of Cα atoms for residues Gly327 (the equivalent residue in IgG1 is Ala) and Pro329 were altered by approximately 9.9 and 6.7 Å, respectively, compared with their positions in IgG1-Fc (*Fig.* 8*B*). In IgG4-Fc, the different conformation folded the Cγ2 FG loop away from the Cγ2 domain (*Fig.* 8*A*), with the implication that it would disrupt the hydrophobic proline sandwich interaction with receptor, contributing to a loss of approximately 150 Å^2^ total buried surface area from the interface (*Fig.* 9*A*).

The conformation of the Cγ2 BC loop is generally conserved in IgG1-Fc, in which Asn325 from the FG loop can form hydrogen bonds with backbone atoms of Asp270 and Glu272 from the BC loop, and additional van der Waals interactions (*Fig.* 8*C*), although one complex between IgG1-Fc and FcγRI is an exception (55), which we will address in a later section. On the other hand, in IgG4-Fc, the Pro271 side chain is rotated toward the conformationally altered FG loop, the result of a *trans* (IgG1)/*cis* (IgG4) proline isomerization, causing a rearrangement in the hydrogen bond formed by Asn325, creating a new bond with the Pro271 backbone (*Fig.* 8*D*).

The Cγ2 domain BC and FG loops are not only important for FcγR engagement but are additionally involved in C1q binding, an early step in activating the complement cascade through the classical pathway (24,25,71). There is currently no crystal structure available for an IgG-Fc/C1q complex, but residues Asp270 (BC loop), Lys322 (F strand), Pro329 (FG loop), and Pro331 in IgG1 and IgG3 (FG loop) have been implicated in C1q binding (24,25,71). In IgG4, the Ser331Pro mutation partially restores C1q binding, while the reciprocal mutation in IgG1 and IgG3 leads to a reduction (24,25). The altered BC and FG loop conformations in the high-resolution IgG4-Fc crystal structures thus disrupt both FcγR and C1q binding sites (*Fig.* 9*A–C*).

### A distinctive sequence defines the unique IgG4 Cγ2 FG loop

We were keen to determine whether the unexpected IgG4 Cγ2 FG loop conformation existed in any other antibody isotype, but found that it was broadly conserved. In IgG1 and IgE, structural conservation is consistent with the role of this loop in FcγR and FcεRI engagement, respectively, through the hydrophobic proline sandwich.

**Fig. 10:**
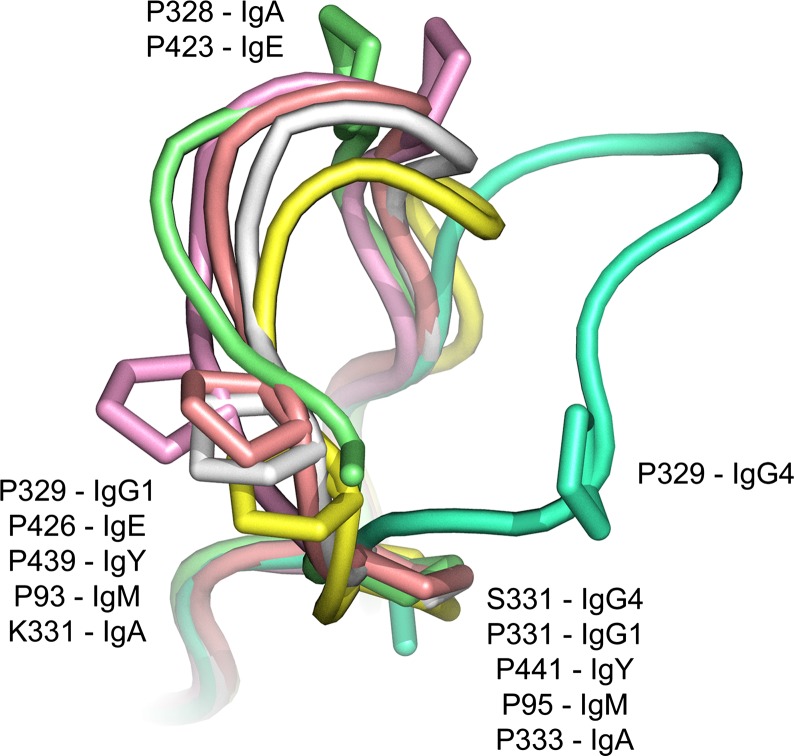
The IgG4 Cγ2 FG loop conformation is unique. The IgG1 Cγ2 FG loop (white) (50), IgE Cε3 FG loop (pink) (82), IgY Cυ3 FG loop (salmon) (153), IgM Cμ3 FG loop (yellow) (160), and IgA Cα2 FG loop (light green) (156) adopt a conserved conformation. The IgG4 Cγ2 FG loop conformation (dark green) (18), which contains a single proline residue at position 329, is unique. Residue numbering is according to the Protein Data Bank entry for each structure. The figure was prepared with PyMOL (167).

Like IgE, an extra Cυ2 domain pair replaces the antibody hinge region in IgY, an isotype found in reptiles and birds. IgY also engages multiple receptors, CHIR-AB1 at the Cυ3-4 interface (149,150), FcRY with the Cυ4 domain (150) and ggFcR, the binding site which utilizes the Cυ3 FG loop (152). The conformation of the IgY Cυ3 FG loop is conserved (153), and like IgG and IgE, a proline residue is found in a structurally equivalent position to Pro329 in IgG, and Pro426 in IgE. Unlike IgG, IgE, and IgY, IgA does not engage receptors using the hinge proximal domain of its Cα2 domain, instead utilizing the Cα3 domain and Cα2–Cα3 domain interface to engage FcαRI, Fcα/μR, and pIgR (154–157). Remarkably, while the sequence of the Cα2 FG loop is not conserved, the receptor-binding proline residue substituted for lysine, the conformation is still similar to that found in IgG1 and IgE. Less is known about the interactions between IgM and its receptors, Fcα/μR, pIgR, and FcμR, although pIgR and Fcα/μR bind the Cμ3-4 fragment (155,157–159), but the Cμ3 FG loop structure still appears to be conserved (160). Human IgG4-Fc is the only subclass in which the altered, unique Cγ2 FG loop conformation has been observed thus far (*Fig.* 10).

A distinguishing feature of isotypes and subclasses in which the Cγ2 FG loop conformation is conserved, whether or not it engages receptor, is that its sequence contains two proline residues. In IgG1, Pro329 engages receptor, while a second proline residue is found at position 331; there are no crystal structures available for IgG3-Fc, but the FG loop sequence is identical to IgG1 and would thus be expected to adopt a similar conformation. IgM and IgY both contain structurally equivalent proline residues to Pro329 and Pro331 from IgG1. In IgE, a second proline residue is found at position 423, in addition to the receptor-engaging Pro426. On the other hand, while the IgA Cα2 FG loop does not contain a receptor-binding proline residue, proline residues are found in a structurally equivalent position to Pro331 in IgG, and Pro423 in IgE (*Fig.* 10). The human IgD Cδ2 domain FG loop not only contains proline residues equivalent to Pro423 and Pro426 in IgE, but a third proline at position 427 (position 330 in IgG), although the structural consequences for the FG loop conformation are unknown.

IgG4 is thus unique among human antibodies in that its Cγ2 FG loop contains a single proline residue, at position 329. Furthermore, a glycine residue is found at position 327 in IgG4, in contrast to an alanine in IgG1, which, together with Ser331, introduces the potential for conformational flexibility. Intriguingly, the human IgG2 FG loop contains sequence elements of both IgG1 (residue 331 is proline) and IgG4 (residue 327 is glycine). Akin to human IgG2, the Cγ2 FG loop of some non-human primate IgG subclasses also contain glycine at position 327, but to the best of our knowledge, Pro329 and Pro331 are conserved (160,161).

Three crystal structures have been solved for human IgG2-Fc (72,73). In two structures (72), the Cγ2 FG loop adopted an IgG1-like conformation, but it is important to note that crystal packing precluded an IgG4-like conformation. A similar, unperturbed FG loop conformation was found in an IgG1 Pro331Ser mutant (68), but again crystal packing precluded any conformational change. On the other hand, in a crystal structure of an IgG2-Fc mutant, in which the IgG4 FG loop was created through two point mutations, Ala330Ser and Pro331Ser, the FG loop adopted an IgG4-like conformation (73). It is not yet clear how sequence variation in the Cγ2 FG loop, which is critical for the FcγR interaction, impacts on loop conformation and flexibility among IgG subclass members.

### Deglycosylated IgG4-Fc – adding complexity to our understanding of IgG4 Cγ2 domain structure

**Fig. 11:**
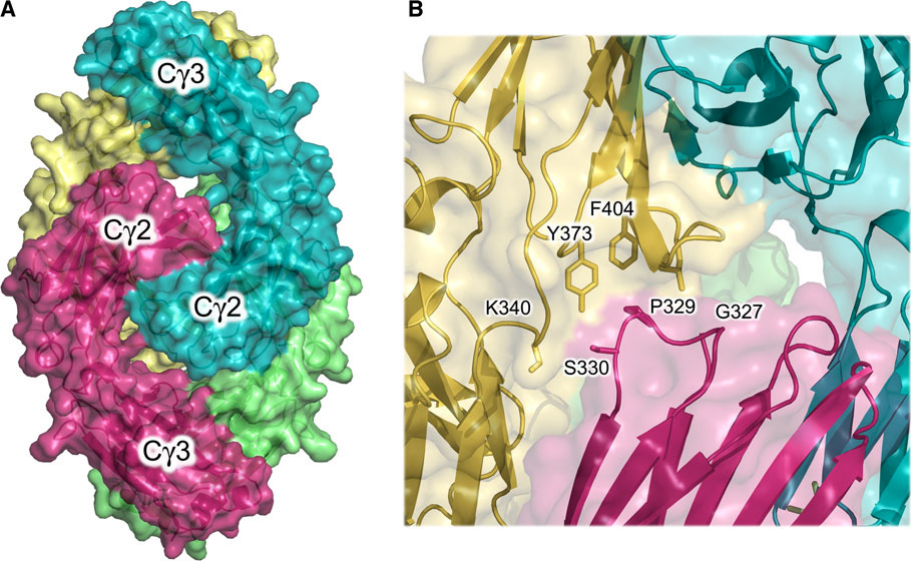
Deglycosylated IgG4-Fc crystal structure. (A) Two IgG4-Fc molecules (blue/yellow and green/pink) form an interlocked arrangement. (B) The Cγ2 domain FG loop forms crystal packing interactions and in the molecule colored pink, adopts the conserved conformation found in IgG1. The figure was prepared with PyMOL (167).

We also solved the 2.7 Å resolution crystal structure of deglycosylated IgG4-Fc, revealing a novel interlocked arrangement of two Fc molecules (19) (*Fig.* 11*A*), in which the Cγ2 domain FG loop formed crystal packing interactions with the Cγ2–Cγ3 domain linker and Cγ3 domain from a neighboring chain (*Fig.* 11*B*). Although partially disordered, in one chain the FG loop adopted the unique conformation observed in IgG4-Fc. On the other hand, the loop adopted the conserved IgG1-like conformation in the other three chains. By contrast, the BC loop exclusively adopted the conserved conformation found in IgG1-Fc. Even though the effects of crystal packing in the deglycosylated IgG4-Fc structure cannot be disregarded, the ability of the BC and FG loops to adopt two different structures does provide evidence for their conformational flexibility in IgG4.

### Cγ2 domain BC loop flexibility

**Fig. 12:**
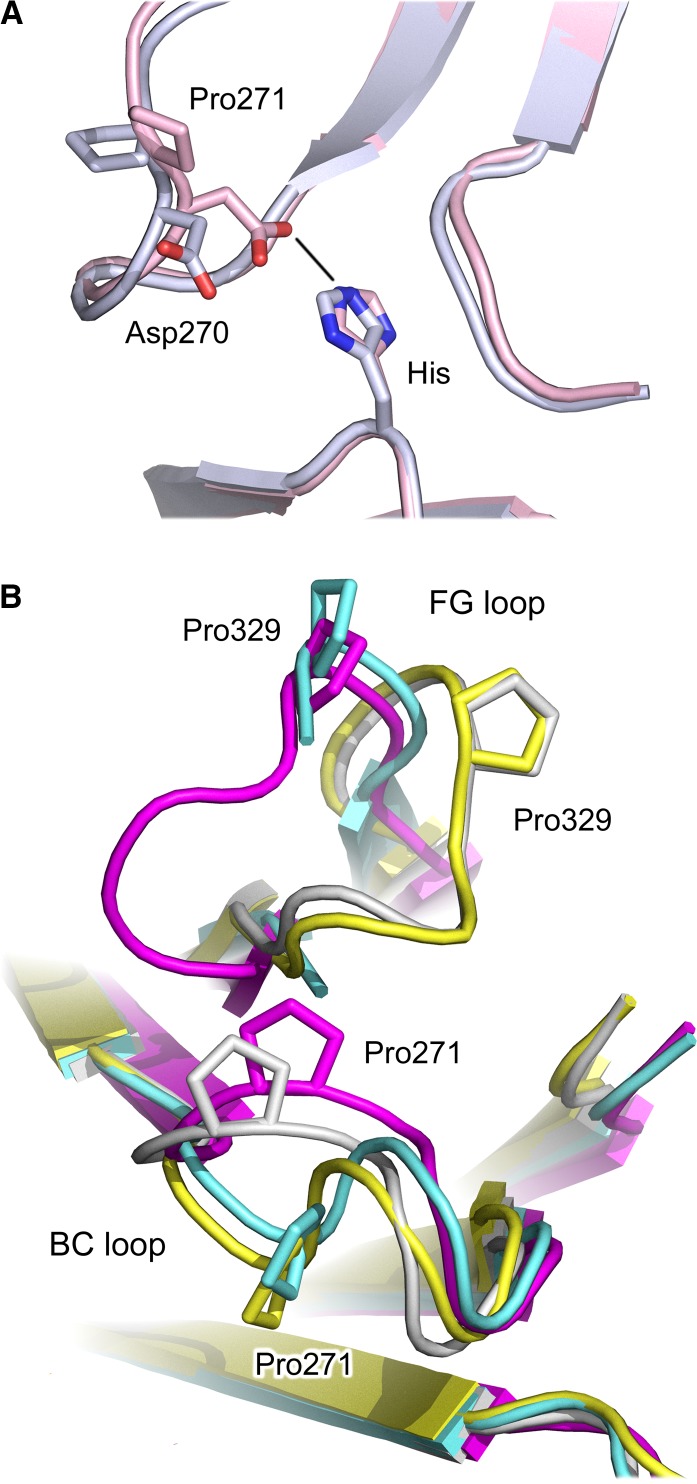
Cγ2 domain loop conformations. (A) In the IgG1-Fc/FcγRIIIa complex (light blue) (58), the conserved Cγ2 BC loop conformation precludes hydrogen bond formation between Asp270 (IgG1 Cγ2 BC loop) and a histidine residue from the receptor. In one IgG1-Fc/FcγRI complex (pink) (55), Pro271 isomerization alters the conformation of the BC loop, permitting hydrogen bond formation. (B) Four different combinations of Cγ2 BC and FG loop conformations are possible: yellow – conserved BC and FG loop, found in non-receptor-bound human IgG1 (e.g. (50)), blue – conserved BC loop and unique FG loop, found in deglycosylated IgG4-Fc (19), gray – non-conserved BC loop and conserved FG loop, found in one IgG1-Fc/FcγRI complex (55), purple – non-conserved BC loop and unique FG loop, found in IgG4-Fc (18). The figure was prepared with PyMOL (167).

Unlike the altered Cγ2 FG loop conformation, which to date appears to be unique to IgG4, the altered Cγ2 BC loop conformation is not unique to this subclass. To the best of our knowledge, the BC loop conformation is conserved in wildtype IgG1-Fc structures (with one exception, discussed below), but differences have been noted in IgG1-Fc fragments mutated to modify effector function and receptor affinity (64,65). We note that in one of the recent IgG1-Fc/FcγRI complexes, the BC loop actually adopts an IgG4-like conformation, i.e. a *trans* (IgG1) to *cis* (IgG4) isomerization of Pro271 in the Cγ2 domain, which engages the receptor D2 domain (55). Pro271 isomerization allows a hydrogen bond to form between Asp270 and a histidine residue from the receptor, an interaction that is precluded by the conserved BC loop conformation (*Fig.* 12*A*). However, both FG loops, and the BC loop from the other Cγ2 domain, are unaltered.

Four different combinations of BC and FG loop conformations are thus possible (*Fig.* 12*B*): (i) conserved BC and FG loop, found in non-receptor-bound human IgG1-Fc; (ii) non-conserved BC loop and conserved FG loop, found in one IgG1-Fc/FcγRI complex; (iii) conserved BC loop and unique FG loop, found in deglycosylated IgG4-Fc; and (iv) non-conserved BC loop and unique FG loop, found in the high-resolution IgG4-Fc structures.

We initially thought that the unique IgG4 Cγ2 FG loop conformation had caused the altered BC loop structure, while others instead proposed that loss of an electrostatic interaction between His268 (BC loop) and Glu294, based on the structure of a His268Ala substitution in a mutant IgG2-Fc molecule, was responsible (73). It now appears that the Cγ2 BC and FG loop conformations are independent of one another, but could be sensitive to their local environment (e.g. when interacting with a high-affinity FcγR). However, the IgG4 Cγ2 BC loop could be predisposed to undergo Pro271 isomerization, due to the unique Cγ2 FG loop conformation.

## Intact IgG4 structure

At the time of writing, no crystal structures are available for intact IgG4. Earlier solution studies suggested a more compact ‘T’-shaped structure for IgG4, with the Fabs positioned close to the Fc region (46). X-ray and neutron scattering solution structures by Rayner *et al*. (44) for wildtype IgG4, and a Ser228Pro mutant which prevents FAE, revealed a largely asymmetric IgG4 structure, particularly at higher protein concentrations, although some symmetry was reported at lower protein concentrations. Despite a hinge with an extended conformation, the Fabs were oriented close to the Fc region, and it was suggested that the shorter IgG4 hinge could limit the conformational freedom of the Fabs, and sterically interfere with C1q binding, but still allow FcγR engagement (44). A different study of a Ser228Pro IgG4 mutant also suggested that the C1q binding site could be obstructed by the Fabs (48). By contrast, the FcγR binding site and proposed C1q binding site were not occluded by the Fabs in the asymmetric IgG1 solution structures (47).

In a different study, a symmetric structure was reported for wildtype IgG4, whereas the conformation of a Ser331Pro mutant was asymmetric (41). Given that the IgG1-Fc Cγ2 domain FG loop forms van der Waals contacts with the junction between the lower hinge and Cγ2 domain, and additionally contacts one Fab in the human IgG1 crystal structure (36), it is possible this flexible loop, which can adopt different conformations, could influence overall IgG4 conformation, and the disposition of Fab fragments relative to the Fc.

## IgG4 structure – implications for C1q binding

IgG Fc–Fc-mediated hexameric assemblies play a crucial role in complement activation, in which C1q binds to one face of the hexamer (90). The disrupted C1q binding site (*Fig.* 9*C*) is consistent with the inability of intact IgG4 to activate complement (3,24,163); moreover, docking of the C1q structure onto solution structures suggests that the Fabs might sterically interfere with the IgG4-Fc/C1q interaction (44).

On the other hand, IgG4-Fc is able to bind C1 (164). The removal of any steric impediment from the Fabs, and the ability of the IgG4 Cγ2 BC and FG loops to adopt a conserved IgG1-like conformation support this observation. Furthermore, IgG4 is able to form a hexameric assembly, essentially identical to that for IgG1 (19,36) (*Fig.* 2*H*).

A Glu345Arg mutation at the Cγ2–Cγ3 interface, which presumably enhances contact between Fc molecules in the hexamer, was found to enhance complement activation in all IgG subclasses (90). Intriguingly, the mutation is distal to the residues known to contribute to the IgG-Fc C1q binding site (165), and implies that enhanced hexamer formation in IgG4 can overcome both disruption of the C1q binding site, and steric hindrance by the Fabs. A crystal structure of IgG-Fc in complex with C1q would undoubtedly shed further light on this fascinating observation.

## IgG4-Fc structure – speculation about the interaction with FcγRs

Our current understanding of the structural basis for the interaction with FcγRs is entirely dependent on complexes with IgG1-Fc (54–60,64,65), yet we still lack a complete understanding of the interaction even for this subclass. Only one low-resolution crystal structure is available for an FcγRIIa complex (60), FcγRIIb complexes are with IgG1-Fc mutants which have selectively enhanced affinity for this receptor (65), and the two FcγRI structures differ in detail in their interaction with IgG1-Fc (54,55). There are no crystal structures for any other subclass in complex with an FcγR, which could reveal additional diversity in the interaction between IgG and its receptors.

The IgG4-Fc crystal structures reveal Cγ2 domain loop conformations that would clearly have an impact on the interaction between this subclass and FcγRs. In this section, we speculate about the potential consequences of the unique structure of IgG4 for FcγR interactions, and how it might relate to receptor affinity. We consider the two binding sites affected by Cγ2 loop conformations, namely the proline sandwich and the interface with the receptor D2 domain in turn, in addition to the interface with the lower hinge.

### The proline sandwich

A distinguishing feature of the IgG4-Fc crystal structure is the altered Cγ2 domain FG loop conformation which would disrupt the hydrophobic proline sandwich interaction (*Fig.* 9*A*). What would be the implications for the interaction between IgG4 and FcγRs if this loop were to adopt the unique conformation?

**Fig. 13:**
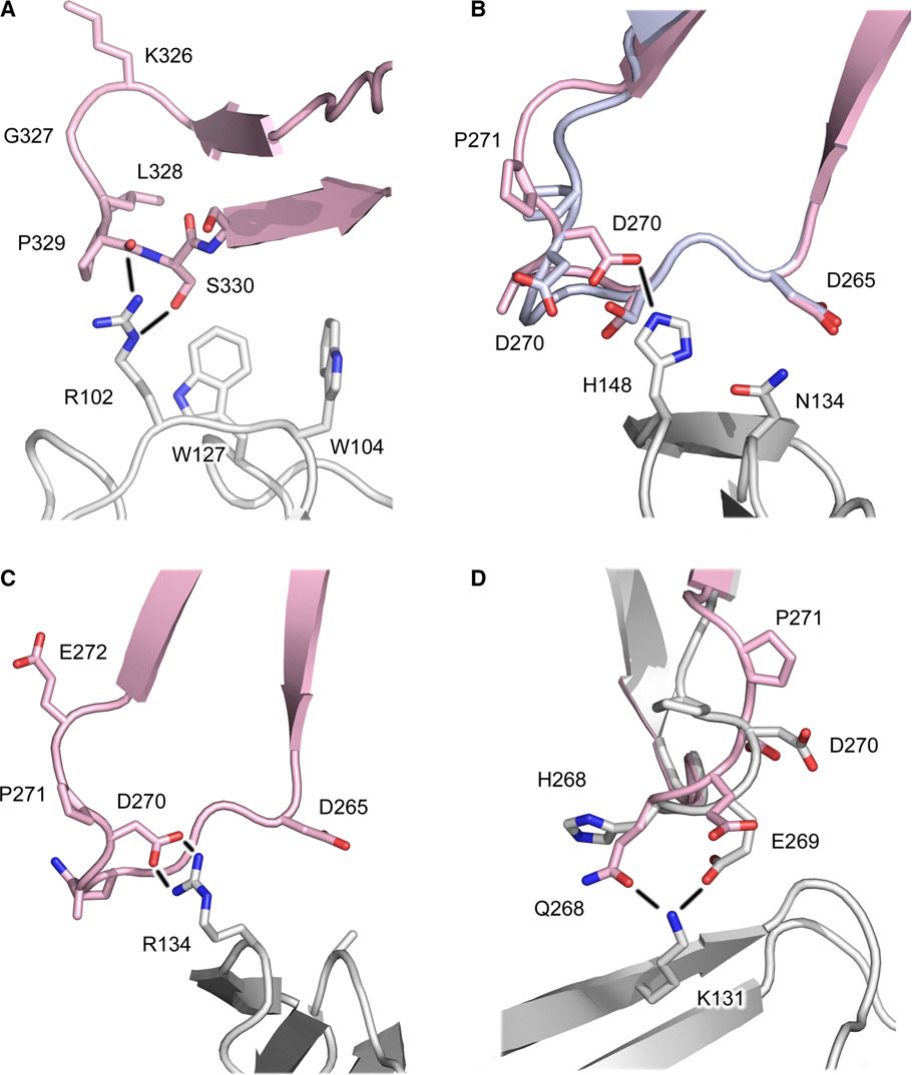
Potential interactions between IgG4-Fc and FcγRs. (A) The IgG4 Cγ2 domain FG loop (pink) (18) disrupts the hydrophobic proline sandwich. The Pro329 carbonyl oxygen atom and the Ser330 side chain could form hydrogen bonds, indicated by black lines, with Arg102 from FcγRI (white) (55). Of the human IgG subclasses, Ser330 is unique to IgG4. (B) The conserved Cγ2 BC loop conformation found in IgG1 (light blue) (55) precludes hydrogen bond formation between Asp270 and His148 from FcγRI. The Cγ2 BC loop conformation in IgG4 (pink) (18), in which Pro271 undergoes a *cis*/*trans* isomerization, would enable Asp270 to form a hydrogen bond with His148 from FcγRI (white) (55). In one FcγRI complex structure, Pro271 from the IgG1 Cγ2 BC loop also undergoes a *cis/trans* isomerization (55). (C) The same *cis/trans* isomerization would enable Asp270 from the IgG4 Cγ2 BC loop (pink) (18) to form a salt bridge with Arg134 from FcγRIIb (white) (65). (D) In the IgG1-Fc/FcγRIIIa complex (white) (58), Glu269 from the Cγ2 BC loop forms a hydrogen bond with Lys131. Gln268 from the IgG4 Cγ2 BC loop (pink) (18) would also be able to form a hydrogen bond with Lys131. The figure was prepared with PyMOL (167).

A position adjacent to the proline sandwich is a site of sequence variation between FcγRs, in which structurally equivalent residues are arginine in FcγRI (Arg102), serine in FcγRII (Ser88), and isoleucine in FcγRIII (Ile88). Modeling based on the FcγRI structures suggests that Arg102 could form two hydrogen bonds with the IgG4 Cγ2 FG loop – one with the Pro329 backbone carbonyl group, and one with the Ser330 side chain (*Fig.* 13*A*). Thus, although the proline sandwich interaction would be disrupted, one additional hydrogen bond could form in an IgG4/FcγRI complex, compared with that for IgG1, in which one hydrogen bond already forms between Arg102 from the receptor and the Pro329 carbonyl group. These hydrogen bonds would not be possible in FcγRII or FcγRIII complexes, for which IgG4 has lower affinity (98), in which Arg102 is replaced by serine and isoleucine, respectively. Furthermore, of the four IgG subclasses, Ser330 is unique to IgG4.

An IgG4 Phe234Leu/Ser331Pro mutant, which renders the IgG4 Cγ2 FG loop more IgG1-like, did not lead to an enhancement in affinity for FcγRI, compared with an IgG4 Phe234Leu mutant alone (21), implying that a disrupted Cγ2 FG loop, with serine uniquely at position 330, could still contribute towards a high-affinity interaction with this receptor.

It is also quite possible that a conserved, IgG1-like, IgG4 Cγ2 FG loop conformation engages receptor; the deglycosylated IgG4-Fc crystal structure provides evidence for adoption of this IgG1-like conformation. However, the distribution of conformations of the Cγ2 FG loop in solution is unknown. If the FG loop is dynamic, preferring no single conformation, but engages receptor in a conserved manner, ordering of the loop upon formation of the hydrophobic proline sandwich interaction could affect the net entropic contribution to receptor binding. Indeed, different enthalpic and entropic contributions to binding of different FcγRs have been reported for IgG1 (110,111).

The disrupted proline sandwich could contribute to the lower affinity of IgG4 for particular FcγRs, compared with IgG1 (98), but lower affinity could also be attributed to other determinants, such as substitution of the lower hinge residue Leu234 in IgG1, to Phe234 in IgG4 (21) and the overall disposition of the Fabs (44,48).

### Receptor D2 domain

The receptor D2 domain, which engages the second IgG-Fc Cγ2 domain, displays greater sequence variation between FcγR classes and sub-types, compared with the conserved tryptophan residues forming the hydrophobic proline sandwich interaction. A prominent feature of this interface is the interaction with the IgG-Fc Cγ2 BC loop.

In the majority of FcγR complex structures, the position of the IgG1 Cγ2 BC loop precludes any contact with residue 148 (FcγRI)/134 (FcγRII and FcγRIII) from the receptor, and this interaction would be similarly precluded for the conserved IgG4 Cγ2 BC loop conformation, found in the deglycosylated IgG4-Fc structure (19). The identity of residue 148/134 varies between different receptors (histidine in FcγRI and FcγRIIIa/b, arginine in FcγRIIb) and is the site of an Arg/His polymorphism in FcγRIIa.

In the FcγRI complex structure solved by Kiyoshi *et al*. (55), a hydrogen bond between Asp270 from the IgG1 Cγ2 BC loop and His148 from the receptor was noted. Formation of this hydrogen bond is facilitated by the same *cis*/*trans* isomerization of Pro271 found in the IgG4-Fc crystal structures (18). Crystal structures for mutant IgG1-Fc molecules in complex with FcγRIIb and FcγRIIIa reveal similar conformations in the Cγ2 BC loop, in which a hydrogen bond forms with His134 from FcγRIIIa (64), and a salt bridge with Arg134 from FcγRIIb (65). It is noteworthy that these mutated IgG1-Fc regions displayed either enhanced affinity for FcγRIIa and FcγRIIIa, or selectively enhanced affinity for FcγRIIb over both FcγRIIa polymorphic variants.

Isomerization of Pro271 in one IgG1-Fc/FcγRI complex (55), and the structural changes associated with IgG1-Fc mutations conferring enhanced affinity (64,65), link Cγ2 BC loop conformation with changes in FcγR affinity. The IgG4 Cγ2 BC loop could play the same role in modulating receptor affinity. Asp270 would be ideally placed to form hydrogen bonds with His148/134 from FcγRI and FcγRIIIa, and the His134 FcγRIIa polymorph, and a salt bridge with Arg134 from FcγRIIb and the Arg134 FcγRIIa polymorph (*Fig.* 13*B, C*). This interaction could account for the higher affinity of IgG4 for the Arg134 FcγRIIa polymorph, over the His134 FcγRIIa polymorph, and contribute to the interaction with FcγRIIb, the affinity for which is the highest of all IgG subclasses (98).

Although the position of the IgG-Fc Cγ2 domain is not identical when the IgG1-Fc/FcγR complexes are superposed on the receptor D2 domain, in a number of structures the C′ strand residue Lys145 (FcγRI)/131 (FcγRII and FcγRIII) forms a hydrogen bond with Glu269 from the IgG1-Fc Cγ2 BC loop (54,56,58), and in one FcγRI structure (55), a water-mediated hydrogen bond. As the *cis*/*trans* isomerization of Pro271 in the BC loop does not alter the position of the Glu269 Cα atom (and side chain) so as to preclude hydrogen formation with Lys145/131, IgG4 would also be capable of forming a hydrogen bond with this invariant lysine residue (*Fig.* 13*D*). However, residue 268 from the BC loop is histidine in IgG1 and glutamine in IgG4; Gln268, with its greater conformational flexibility, would also be able to form a hydrogen bond with Lys145/131, and could affect the interaction with Glu269.

### The lower hinge

The IgG4 hinge region is three residues shorter than its counterpart in IgG1, and the lower hinge, positioned closest to the Fc region, differs at a single position (*Fig.* 6*D*). In IgG1, residue 234 is leucine, whereas in IgG4, this residue is phenylalanine. The Phe234Leu mutation enhances the affinity of IgG4 for FcγRI (21). The hinge region was disordered in our IgG4-Fc crystal structures, and is likewise not fully ordered in some IgG1-Fc/FcγR complex structures. We modeled a Leu234Phe mutation in some complex structures, and found that this residue could be accommodated in all three FcγR complexes, but its conformational flexibility would be more restricted than leucine at this position, particularly in the IgG-Fc chain which interacts with the receptor D2 BC and FG loops. On the other hand, occupation of the hydrophobic pocket by Leu235, and the interaction with the receptor D2 FG loop, determinants of high-affinity FcγRI binding (32,55,166), would be expected to be similar to IgG1.

## Concluding remarks and future directions

IgG4 is an intriguing antibody with unique biological properties, and the interaction between IgG4 and FcγRs plays an important role in disease mechanisms. Despite evidence for impairing anti-tumor immunity in malignant melanoma, and the ability to inhibit mast cell degranulation in allergy, the molecular basis for the role of IgG4 in disease, whether deleterious or beneficial, is not fully understood. Functional studies would clarify the mechanisms by which IgG4 exerts its protective effects in allergic disease, potentially through interactions with FcγRIIb. Furthermore, it is fascinating that despite affinities which are of the same order of magnitude, engagement of the same receptor, FcγRI, by IgG1 and IgG4 can produce such different outcomes in anti-tumor responses. Crystal structures would shed light on whether there are fundamental differences between IgG1 and IgG4 FcγRI complexes, perhaps involving the orientation of the receptor D3 domain, or the disposition of the Fabs (affected by different hinge positions), which could account for the different ‘signal’ received by the effector cell.

Until recently, only one low-resolution crystal structure was available for IgG4-Fc. High-resolution crystal structures for IgG4-Fc revealed a unique conformation in the Cγ2 domain FG loop which would be expected to disrupt, or at least alter, the interaction with FcγRs, raising questions about how IgG4 engages receptors. Could IgG4 once again ‘break the rules’ and engage certain FcγRs in a non-conserved manner, without utilizing the conserved proline sandwich? What is the molecular basis for the unique pattern of binding affinities between IgG4 and FcγRs? At the moment we can only speculate upon the structural details of these interactions, but as crystal structures become available for IgG4, and indeed other subclasses, we will undoubtedly gain a deeper understanding of the complex relationship between the four IgG subclasses and their FcγRs.

## References

[1] Aalberse RC, Stapel SO, Schuurman J, Rispens T (2009). Immunoglobulin G4: an odd antibody. Clin Exp Allergy.

[2] Aalberse RC, Schuurman J (2002). IgG4 breaking the rules. Immunology.

[3] van der Zee JS, van Swieten P, Aalberse RC (1986). Inhibition of complement activation by IgG4 antibodies. Clin Exp Immunol.

[4] van der Zee JS, van Swieten P, Aalberse RC (1986). Serologic aspects of IgG4 antibodies. II. IgG4 antibodies form small, nonprecipitating immune complexes due to functional monovalency. J Immunol.

[5] Salfeld JG (2007). Isotype selection in antibody engineering. Nat Biotechnol.

[6] Jiang X-R (2011). Advances in the assessment and control of the effector functions of therapeutic antibodies. Nat Rev Drug Discov.

[7] Brennan FR (2010). Safety and immunotoxicity assessment of immunomodulatory monoclonal antibodies. MAbs.

[8] Persselin JE, Stevens RH (1988). Serum IgG4 anti-Fab antibodies in rheumatoid arthritis are constitutively expressed. Rheumatol Int.

[9] Chen L-F, Mo Y-Q, Ma J-D, Luo L, Zheng D-H, Dai L (2014). Elevated serum IgG4 defines specific clinical phenotype of rheumatoid arthritis. Mediators Inflamm.

[10] Chapuy-Regaud S, Nogueira L, Clavel C, Sebbag M, Vincent C, Serre G (2005). IgG subclass distribution of the rheumatoid arthritis-specific autoantibodies to citrullinated fibrin. Clin Exp Immunol.

[11] Cohen PL, Cheek RL, Hadler JA, Yount WJ, Eisenberg RA (1987). The subclass distribution of human IgG rheumatoid factor. J Immunol.

[12] van de Stadt LA (2014). Antibodies to IgG4 hinge can be found in rheumatoid arthritis patients during all stages of disease and may exacerbate chronic antibody-mediated inflammation. Arthritis Rheum.

[13] Stone JH, Zen Y, Deshpande V (2012). IgG4-related disease. N Engl J Med.

[14] Pieringer H, Parzer I, Wöhrer A, Reis P, Oppl B, Zwerina J (2014). IgG4- related disease: an orphan disease with many faces. Orphanet J Rare Dis.

[15] Karagiannis P (2013). IgG4 subclass antibodies impair antitumor immunity in melanoma. J Clin Invest.

[16] Santos AF (2015). IgG_4_ inhibits peanut-induced basophil and mast cell activation in peanut-tolerant children sensitized to peanut major allergens. J Allergy Clin Immunol.

[17] Davies AM (2013). Crystal structure of the human IgG4 C_H_3 dimer reveals the role of Arg409 in the mechanism of Fab-arm exchange. Mol Immunol.

[18] Davies AM (2014). Structural determinants of unique properties of human IgG4-Fc. J Mol Biol.

[19] Davies AM, Jefferis R, Sutton BJ (2014). Crystal structure of deglycosylated human IgG4-Fc. Mol Immunol.

[20] Vidarsson G, Dekkers G, Rispens T (2014). IgG subclasses and allotypes: from structure to effector functions. Front Immunol.

[21] Canfield SM, Morrison SL (1991). The binding affinity of human IgG for its high affinity Fc receptor is determined by multiple amino acids in the CH2 domain and is modulated by the hinge region. J Exp Med.

[22] van der Neut Kolfschoten M (2007). Anti-inflammatory activity of human IgG4 antibodies by dynamic Fab arm exchange. Science.

[23] Labrijn AF (2011). Species-specific determinants in the IgG CH3 domain enable Fab-arm exchange by affecting the noncovalent CH3-CH3 interaction strength. J Immunol.

[24] Tao MH, Smith RI, Morrison SL (1993). Structural features of human immunoglobulin G that determine isotype-specific differences in complement activation. J Exp Med.

[25] Xu Y, Oomen R, Klein MH (1994). Residue at position 331 in the IgG1 and IgG4 CH2 domains contributes to their differential ability to bind and activate complement. J Biol Chem.

[26] Jefferis R (1990). A comparative study of the N-linked oligosaccharide structures of human IgG subclass proteins. Biochem J.

[27] Niwa R (2005). IgG subclass-independent improvement of antibody-dependent cellular cytotoxicity by fucose removal from Asn^297^-linked oligosaccharides. J Immunol Methods.

[28] Pincetic A, Maamary J, Ravetch JV, Taniguchi N, Endo T, Hart GW, Seeberger PH, Wong C-H (2015). Therapeutic applications of sialylated IVIG. Glycoscience: Biology and Medicine.

[29] Raju TS (2008). Terminal sugars of Fc glycans influence antibody effector functions of IgGs. Curr Opin Immunol.

[30] Anthony RM, Ravetch JV (2010). A novel role for the IgG Fc Glycan: the anti-inflammatory activity of sialylated IgG Fcs. J Clin Immunol.

[31] Kaneko Y, Nimmerjahn F, Ravetch JV (2006). Anti-inflammatory activity of immunoglobulin G resulting from Fc sialylation. Science.

[32] Lu J, Ellsworth JL, Hamacher N, Oak SW, Sun PD (2011). Crystal structure of Fcγ receptor I and its implication in high affinity γ-immunoglobulin binding. J Biol Chem.

[33] Malhotra R, Wormald MR, Rudd PM, Fischer PB, Dwek RA, Sim RB (1995). Glycosylation changes of IgG associated with rheumatoid arthritis can activate complement via the mannose-binding protein. Nat Med.

[34] Parekh RB (1985). Association of rheumatoid arthritis and primary osteoarthritis with changes in the glycosylation pattern of total serum IgG. Nature.

[35] Ercan A (2010). Aberrant IgG galactosylation precedes disease onset, correlates with disease activity, and is prevalent in autoantibodies in rheumatoid arthritis. Arthritis Rheum.

[36] Saphire EO (2001). Crystal structure of a neutralizing human IgG against HIV-1: a template for vaccine design. Science.

[37] Wu Y, West AP, Kim HJ, Thornton ME, Ward AB, Bjorkman PJ (2013). Structural basis for enhanced HIV-1 neutralization by a dimeric immunoglobulin G form of the glycan-recognizing antibody 2G12. Cell Rep.

[38] Harris LJ, Larson SB, Hasel KW, McPherson A (1997). Refined structure of an intact IgG2a monoclonal antibody. Biochemistry.

[39] Harris LJ, Skaletsky E, McPherson A (1998). Crystallographic structure of an intact IgG1 monoclonal antibody. J Mol Biol.

[40] Sandin S, Öfverstedt L-G, Wikström A-C, Wrange Ö, Skoglund U (2004). Structure and flexibility of individual immunoglobulin G molecules in solution. Structure.

[41] Lu Y (2007). Solution conformation of wild-type and mutant IgG3 and IgG4 immunoglobulins using crystallohydrodynamics: possible implications for complement activation. Biophys J.

[42] Rayner LE, Kadkhodayi-Kholghi N, Heenan RK, Gor J, Dalby PA, Perkins SJ (2013). The solution structure of rabbit IgG accounts for its interactions with the Fc receptor and complement C1q and its conformational stability. J Mol Biol.

[43] Abe Y, Gor J, Bracewell DG, Perkins SJ, Dalby PA (2010). Masking of the Fc region is human IgG4 by constrained X-ray scattering modeling: implications for antibody function and therapy. Biochem J.

[44] Rayner LE, Hui GK, Gor J, Heenan RK, Dalby PA, Perkins SJ (2014). The Fab conformations in the solution structure of human immunoglobulin G4 (IgG4) restrict access to its Fc region: implications for functional activity. J Biol Chem.

[45] Lilyestrom WG, Shire S, Scherer TM (2012). Influence of the cosolute environment on IgG solution structure analyzed by small-angle X-ray scattering. J Phys Chem B.

[46] Gregory L (1987). The solution conformations of the subclasses of human IgG deduced from sedimentation and small angle X-ray scattering studies. Mol Immunol.

[47] Rayner LE, Hui GK, Gor J, Heenan RK, Dalby PA, Perkins SJ (2015). The solution structures of two human IgG1 antibodies show conformational stability and accommodate their C1q and FcγR ligands. J Biol Chem.

[48] Tian X, Vestergaard B, Thorolfsson M, Yang Z, Rasmussen HB, Langkilde AE (2015). In-depth analysis of subclass-specific conformational preferences of IgG antibodies. IUCrJ.

[49] Deisenhofer J (1981). Crystallographic refinement and atomic models of a human Fc fragment and its complex with fragment B of protein A from *Staphylococcus aureus* at 2.9- and 2.8-Å resolution. Biochemistry.

[50] Matsumiya S (2011). Corrigendum to “Structural comparison of fucosylated and nonfucosylated Fc fragments of human immunoglobulin G1” [J. Mol. Biol. 386/3 (2007) 767–779]. J Mol Biol.

[51] Duquerroy S (2007). Crystal structure of a human autoimmune complex between IgM rheumatoid factor RF61 and IgG1 Fc reveals a novel epitope and evidence for affinity maturation. J Mol Biol.

[52] Sauer-Eriksson AE, Kleywegt GJ, Uhlén M, Jones TA (1995). Crystal structure of the C2 fragment of streptococcal protein G in complex with the Fc domain of human IgG. Structure.

[53] Sprague ER, Wang C, Baker D, Bjorkman PJ (2006). Crystal structure of the HSV-1 Fc receptor bound to Fc reveals a mechanism for antibody bipolar bridging. PLoS Biol.

[54] Lu J, Chu J, Zou Z, Hamacher NB, Rixon MW, Sun PD (2015). Structure of FcγRI in complex with Fc reveals the importance of glycan recognition for high-affinity IgG binding. Proc Natl Acad Sci USA.

[55] Kiyoshi M (2015). Structural basis for binding of human IgG1 to its high-affinity human receptor FcγRI. Nat Commun.

[56] Radaev S, Motyka S, Fridman W-H, Sautes-Fridman C, Sun PD (2001). The structure of a human type III Fcγ receptor in complex with Fc. J Biol Chem.

[57] Ferrara C (2011). Unique carbohydrate-carbohydrate interactions are required for high affinity binding between FcγRIII and antibodies lacking core fucose. Proc Natl Acad Sci USA.

[58] Mizushima T (2011). Structural basis for improved efficacy of therapeutic antibodies on defucosylation of their Fc glycans. Genes Cells.

[59] Sondermann P, Huber R, Oosthuizen V, Jacob U (2000). The 3.2-Å crystal structure of the human IgG1 Fc fragment–FcγRIII complex. Nature.

[60] Ramsland PA (2011). Structural basis for FcγRIIa recognition of human IgG and formation of inflammatory signaling complexes. J Immunol.

[61] James LC, Keeble AH, Khan Z, Rhodes DA, Trowsdale J (2007). Structural basis for PRYSPRY-mediated tripartite motif (TRIM) protein function. Proc Natl Acad Sci USA.

[62] DeLano WL, Ultsch MH, de Vos AM, Wells JA (2000). Convergent solutions to binding at a protein–protein interface. Science.

[63] Oganesyan V (2014). Structural insights into neonatal Fc receptor-based recycling mechanisms. J Biol Chem.

[64] Mimoto F, Kadono S, Katada H, Igawa T, Kamikawa T, Hattori K (2014). Crystal structure of a novel asymmetrically engineered Fc variant with improved affinity for FcγRs. Mol Immunol.

[65] Mimoto F (2013). Engineered antibody Fc variant with selectively enhanced FcγRIIb binding over both FcγRIIa^R131^ and FcγRIIa^H131^. Protein Eng Des Sel.

[66] Ahmed AA (2014). Structural characterization of anti-inflammatory immunoglobulin G Fc proteins. J Mol Biol.

[67] Crispin M, Yu X, Bowden TA (2013). Crystal structure of sialylated IgG Fc: implications for the mechanism of intravenous immunoglobulin therapy. Proc Natl Acad Sci USA.

[68] Oganesyan V, Gao C, Shirinian L, Wu H, Dall'Acqua WF (2008). Structural characterization of a human Fc fragment engineered for lack of effector functions. Acta Crystallogr.

[69] Krapp S, Mimura Y, Jefferis R, Huber R, Sondermann P (2003). Structural analysis of human IgG-Fc glycoforms reveals a correlation between glycosylation and structural integrity. J Mol Biol.

[70] Idusogie EE (2000). Mapping of the C1q binding site on rituxan, a chimeric antibody with a human IgG1 Fc. J Immunol.

[71] Matsumiya S (2007). Structural comparison of fucosylated and nonfucosylated Fc fragments of human immunoglobulin G1. J Mol Biol.

[72] Teplyakov A, Zhao Y, Malia TJ, Obmolova G, Gilliland GL (2013). IgG2 Fc structure and the dynamic features of the IgG CH_2_–CH_3_ interface. Mol Immunol.

[73] Vafa O (2014). An engineered Fc variant of an IgG eliminates all immune effector functions via structural perturbations. Methods.

[74] Corper AL (1997). Structure of human IgM rheumatoid factor Fab bound to its autoantigen IgG Fc reveals a novel topology of antibody–antigen interaction. Nat Struct Biol.

[75] Feige MJ, Nath S, Catharino SR, Weinfurtner D, Steinbacher S, Buchner J (2009). Structure of the murine unglycosylated IgG1 Fc fragment. J Mol Biol.

[76] Kolenko P, Dohnálek J, Dušková J, Skálová T, Collard R, Hašek J (2009). New insights into intra- and intermolecular interactions of immunoglobulins: crystal structure of mouse IgG2b-Fc at 2.1-Å resolution. Immunology.

[77] Keeble AH, Khan Z, Forster A, James LC (2008). TRIM21 is an IgG receptor that is structurally, thermodynamically, and kinetically conserved. Proc Natl Acad Sci USA.

[78] Burmeister WP, Huber AH, Bjorkman PJ (1994). Crystal structure of the complex of rat neonatal Fc receptor with Fc. Nature.

[79] Martin WL, West AP, Gan L, Bjorkman PJ (2001). Crystal structure at 2.8Å of an FcRn/heterodimeric Fc complex: mechanism of pH-dependent binding. Mol Cell.

[80] Girardi E, Holdom MD, Davies AM, Sutton BJ, Beavil AJ (2009). The crystal structure of rabbit IgG-Fc. Biochem J.

[81] Holdom MD (2011). Conformational changes in IgE contribute to its uniquely slow dissociation rate from receptor FcεRI. Nat Struct Mol Biol.

[82] Dhaliwal B (2012). Crystal structure of IgE bound to its B-cell receptor CD23 reveals a mechanism of reciprocal allosteric inhibition with high affinity receptor FcεRI. Proc Natl Acad Sci USA.

[83] Garman SC, Wurzburg BA, Tarchevskaya SS, Kinet J-P, Jardetzky TS (2000). Structure of the Fc fragment of human IgE bound to its high-affinity receptor FcεRIα. Nature.

[84] Sutton BJ, Davies AM (2015). Structure and dynamics of IgE-receptor interactions: FceRI and CD23/FceRII. Immunol Rev.

[85] Sondermann S, Pincetic A, Maamary J, Lammens K, Ravetch JV (2013). General mechanism for modulating immunoglobulin effector function. Proc Natl Acad Sci USA.

[86] Roopenian DC, Akilesh S (2007). FcRn: the neonatal Fc receptor comes of age. Nat Rev Immunol.

[87] McEwan WA, Mallery DL, Rhodes DA, Trowsdale J, James LC (2011). Intracellular antibody-mediated immunity and the role of TRIM21. BioEssays.

[88] Zack DJ, Stempniak M, Wong AL, Weisbart RH (1995). Localization of an Fc-binding reactivity to the constant region of human IgG4. Implications for the pathogenesis of rheumatoid arthritis. J Immunol.

[89] Kawa S (2008). A novel immunoglobulin-immunoglobulin interaction in autoimmunity. PLoS ONE.

[90] Diebolder CA (2014). Complement is activated by IgG hexamers assembled at the cell surface. Science.

[91] Nordenfelt P (2012). Antibody orientation at bacterial surfaces is related to invasive infection. J Exp Med.

[92] Nimmerjahn F, Ravetch JV (2008). Fcγ receptors as regulators of immune responses. Nat Rev Immunol.

[93] Hogarth PM, Pietersz GA (2012). Fc receptor-targeted therapies for the treatment of inflammation, cancer and beyond. Nat Rev Drug Discov.

[94] Malbec O, Daëron M (2007). The mast cell IgG receptors and their roles in tissue inflammation. Immunol Rev.

[95] Guilliams M, Bruhns P, Saeys Y, Hammad H, Lambrecht BN (2014). The function of Fcγ receptors in dendritic cells and macrophages. Nat Rev Immunol.

[96] Ravetch JV, Bolland S (2001). IgG Fc receptors. Annu Rev Immunol.

[97] Nimmerjahn F, Ravetch JV (2006). Fcγ receptors: old friends and new family members. Immunity.

[98] Bruhns P (2009). Specificity and affinity of human Fcγ receptors and their polymorphic variants for human IgG subclasses. Blood.

[99] Bruhns P (2012). Properties of mouse and human IgG receptors and their contribution to disease models. Blood.

[100] Smith KGC, Clatworthy RM (2010). FcγRIIB in autoimmunity and infection: evolutionary and therapeutic implications. Nat Rev Immunol.

[101] Nimmerjahn F, Ravetch JV (2012). Translating basic mechanisms of IgG effector activity into next generation cancer therapies. Cancer Immun.

[102] Reddy MP (2000). Elimination of Fc receptor-dependent effector functions of a modified IgG4 monoclonal antibody to human CD4. J Immunol.

[103] Jefferis R (2009). Recombinant antibody therapeutics: the impact of glycosylation on mechanisms of action. Trends Pharmacol Sci.

[104] Shields RL (2001). High resolution mapping of the binding site on human IgG1 for FcγRI, FcγRII, FcγRIII, and FcRn and design of IgG1 variants with improved binding to the FcγR. J Biol Chem.

[105] Hayes JM (2014). Fc gamma receptor glycosylation modulates the binding of IgG glycoforms: a requirement for stable antibody interactions. J Proteome Res.

[106] Galon J (1997). Affinity of the interaction between Fc gamma receptor type III (FcγRIII) and monomeric human IgG subclasses. Role of FcγRIII glycosylation. Eur J Immunol.

[107] Radaev S, Sun P (2001). Recognition of immunoglobulins by Fcγ receptors. Mol Immunol.

[108] Hanson QM, Barb AW (2015). A perspective on the structure and receptor binding properties of immunoglobulin G Fc. Biochemistry.

[109] Woof JM, Burton DR (2004). Human antibody–Fc receptor interactions illuminated by crystal structures. Nat Rev Immunol.

[110] Okazaki A (2004). Fucose depletion from human IgG1 oligosaccharide enhances binding enthalpy and association rate between IgG1 and FcγRIIIa. J Mol Biol.

[111] Maenaka K, van der Merwe P, Stuart D, Jones EY, Sondermann P (2001). The human low affinity Fcγ receptors IIa, IIb, and III bind IgG with fast kinetics and distinct thermodynamic properties. J Biol Chem.

[112] Powell MS (1999). Biochemical analysis and crystallisation of FcγRIIa, the low affinity receptor for IgG. Immunol Lett.

[113] Bloom JW, Madanat MS, Marriott D, Wong T, Chan SY (1997). Intrachain disulfide bond in the core hinge region of human IgG4. Prot Sci.

[114] Rispens T, Ooijevaar-de Heer P, Bende O, Aalberse RC (2011). Mechanism of immunoglobulin G4 Fab-arm exchange. J Am Chem Soc.

[115] Schuurman J, Perdok GJ, Gorter AD, Aalberse RC (2001). The inter-heavy chain disulfide bonds of IgG4 are in equilibrium with intra-chain disulfide bonds. Mol Immunol.

[116] Young E, Lock E, Ward DG, Cook A, Harding S, Wallis GLF (2014). Estimation of polyclonal IgG4 hybrids in normal human serum. Immunology.

[117] Rose RJ (2011). Quantitative analysis of the interaction strength and dynamics of human IgG4 half molecules by native mass spectrometry. Structure.

[118] Hamid O (2013). Safety and tumor responses with lambrolizumab (Anti–PD-1) in melanoma. N Engl J Med.

[119] Wang C (2014). In vitro characterization of the anti-PD-1 antibody nivolumab, BMS-936558, and in vivo toxicology in non-human primates. Cancer Immunol Res.

[120] Topalian SL, Drake CG, Pardoll DM (2012). Targeting the PD-1/B7-H1(PD-L1) pathway to activate anti-tumor immunity. Curr Opin Immunol.

[121] Labrijn AF (2009). Therapeutic IgG4 antibodies engage in Fab-arm exchange with endogenous human IgG4 in vivo. Nat Biotechnol.

[122] Angal S (1993). A single amino acid substitution abolishes the heterogeneity of chimeric mouse/human (IgG4) antibody. Mol Immunol.

[123] Shapiro RI (2011). Development and validation of immunoassays to quantify the half-antibody exchange of an IgG4 antibody, natalizumab (Tysabri®) with endogenous IgG4. J Pharm Biomed Anal.

[124] Castro M (2011). Reslizumab for poorly controlled, eosinophilic asthma: a randomized, placebo-controlled study. Am J Respir Crit Care Med.

[125] Ren R, Dao H (2013). Potential role of ixekizumab in the treatment of moderate-to-severe plaque psoriasis. Clin Cosmet Invest Dermatol.

[126] Piper E (2013). A phase II placebo-controlled study of tralokinumab in moderate-to-severe asthma. Eur Respir J.

[127] Ricart AD (2011). Antibody-drug conjugates of calicheamicin derivative: gemtuzumab ozogamicin and inotuzumab ozogamicin. Clin Cancer Res.

[128] Gould HJ, Sutton BJ (2008). IgE in allergy and asthma today. Nat Rev Immunol.

[129] Satoguina JS, Weyand E, Larbi J, Hoerauf A (2005). T regulatory-1 cells induce IgG4 production by B cells: role of IL-10. J Immunol.

[130] Jeannin P, Lecoanet S, Delneste Y, Gauchat J-F, Bonnefoy J-Y (1998). IgE versus IgG4 production can be differentially regulated by IL-10. J Immunol.

[131] Platts-Mills T, Vaughan J, Squillace S, Woodfolk J, Sporik R (2001). Sensitisation, asthma, and a modified Th2 response in children exposed to cat allergen: a population-based cross-sectional study. Lancet.

[132] Jönsson F, Daëron M (2012). Mast cells and company. Front Immunol.

[133] Zhao W, Kepley CL, Morel PA, Okumoto LM, Fukuoka Y, Schwartz LB (2006). FcγRIIa, not FcγRIIb, is constitutively and functionally expressed on skin-derived human mast cells. J Immunol.

[134] Jönsson F (2012). Human FcγRIIA induces anaphylactic and allergic reactions. Blood.

[135] Till SJ, Francis JN, Nouri-Aria K, Durham SR (2004). Mechanisms of immunotherapy. J Allergy Clin Immunol.

[136] Strait RT, Morris SC, Fred D, Finkelman FD (2006). IgG-blocking antibodies inhibit IgE-mediated anaphylaxis in vivo through both antigen interception and FcγRIIb cross-linking. J Clin Invest.

[137] Kepley CL (2004). Co-aggregation of FcγRII with FcεRI on human mast cells inhibits antigen-induced secretion and involves SHIP-Grb2-Dok complexes. J Biol Chem.

[138] James LK (2012). Allergen specificity of IgG(4)-expressing B cells in patients with grass pollen allergy undergoing immunotherapy. J Allergy Clin Immunol.

[139] Dodev TS (2015). Inhibition of allergen-dependent IgE activity by antibodies of the same specificity but different class. Allergy.

[140] Aalberse RC, van der Gaag R, van Leeuwen J (1983). Serologic aspects of IgG4 antibodies. I. Prolonged immunization results in an IgG4-restricted response. J Immunol.

[141] Collins AM, Davies JM (2013). Allergen capture by IgE and IgG. Enhanced cell-binding by allergen multimers: how complex is it?. Immunol Cell Biol.

[142] Linnebacher M, Maletzki C (2012). Tumor-infiltrating B cells: the ignored players in tumor immunology. OncoImmunology.

[143] Karagiannis P, Gilbert AE, Nestle FO, Karagiannis SN (2013). IgG4 antibodies and cancer-associated inflammation: insights into a novel mechanism of immune escape. OncoImmunology.

[144] Germain C, Gnjatic S, Dieu-Nosjean M-C (2015). Tertiary lymphoid structure-associated B cells are key players in anti-tumor immunity. Front Immunol.

[145] Harada K (2012). Significance of immunoglobulin G4 (IgG4)-positive cells in extrahepatic cholangiocarcinoma: molecular mechanism of IgG4 reaction in cancer tissue. Hepatology.

[146] Yoneda M, Inada H, Kanayama K, Shiraishi T (2013). A case of pancreatic ductal adenocarcinoma with marked infiltration with IgG4-positive cells. J Cytol.

[147] Price MA (2011). CSPG4, a potential therapeutic target, facilitates malignant progression of melanoma. Pigment Cell Melanoma Res.

[148] Greenwood J, Clark M, Waldmann H (1993). Structural motifs involved in human IgG antibody effector functions. Eur J Immunol.

[149] Pürzel J, Schmitt R, Viertlboeck BC, Göbel TW (2009). Chicken IgY binds its receptor at the C_H_3/C_H_4 interface similarly as the human IgA:FcαRI interaction. J Immunol.

[150] Taylor AI, Sutton BJ, Calvert RA (2010). Mutations in an avian IgY-Fc fragment reveal the locations of monocyte Fc receptor binding sites. Dev Comp Immunol.

[151] He Y, Bjorkman PJ (2011). Structure of FcRY, an avian immunoglobulin receptor related to mammalian mannose receptors, and its complex with IgY. Proc Natl Acad Sci USA.

[152] Schreiner B, Viertlboeck BC, Göbel TW (2012). A striking example of convergent evolution observed for the ggFcR:IgY interaction closely resembling that of mammalian FcR:IgG. Dev Comp Immunol.

[153] Taylor AI, Fabiane SM, Sutton BJ, Calvert RA (2009). The crystal structure of an avian IgY-Fc fragment reveals conservation with both mammalian IgG and IgE. Biochemistry.

[154] Woof JM, Russell MW (2011). Structure and function relationships in IgA. Mucosal Immunol.

[155] Ghumra A (2009). Structural requirements for the interaction of human IgM and IgA with the human Fcα/μ receptor. Eur J Immunol.

[156] Herr AB, Ballister ER, Bjorkman PJ (2003). Insights into IgA-mediated immune responses from the crystal structures of human FcαRI and its complex with IgA1-Fc. Nature.

[157] Shibuya A, Honda S (2006). Molecular and functional characteristics of the Fcα/μR, a novel Fc receptor for IgM and IgA. Springer Semin Immunopathol.

[158] Klimovich VB (2011). IgM and its receptors: structural and functional aspects. Biochemistry (Mosc).

[159] Kubagawa H (2009). Identity of the elusive IgM Fc receptor (FcμR) in humans. J Exp Med.

[160] Müller R (2013). High-resolution structures of the IgM Fc domains reveal principles of its hexamer formation. Proc Natl Acad Sci USA.

[161] Nguyen DC, Sanghvi R, Scinicariello F, Pulit-Penaloza J, Hill N, Attanasio R (2014). Cynomolgus and pigtail macaque IgG subclasses: characterization of IGHG genes and computational analysis of IgG/Fc receptor binding affinity. Immunogenetics.

[162] Hogarth PM, Anania JC, Wines BD (2014). The FcγR of humans and non-human primates and their interaction with IgG: implications for induction of inflammation, resistance to infection and the use of therapeutic monoclonal antibodies. Curr Top Microbiol Immunol.

[163] Tao MH, Canfield SM, Morrison SL (1991). The differential ability of human IgG1 and IgG4 to activate complement is determined by the COOH-terminal sequence of the CH2 domain. J Exp Med.

[164] Isenman DE, Dorrington KJ, Painter RH (1975). The structure and function of immunoglobulin domains. II. The importance of interchain disulfide bonds and the possible role of molecular flexibility in the interaction between immunoglobulin G and complement. J Immunol.

[165] Schneider S, Zacharias M (2012). Atomic resolution model of the antibody Fc interaction with the complement C1q component. Mol Immunol.

[166] Duncan AR, Woof JM, Partridge LJ, Burton DR, Winter G (1988). Localization of the binding site for the human high-affinity Fc receptor on IgG. Nature.

[167] The PyMOL Molecular Graphics System, Version 1.1.

[168] Brekke OH, Michaelsen TE, Sandlie I (1995). The structural requirements for complement activation by IgG: does it hinge on the hinge?. Immunol Today.

